# Supplementary material for: Experiences of a National Early Warning Score (NEWS) intervention in care homes during the COVID-19 pandemic: a qualitative interview study
Source: BMJ Open. 2021 Jul 27;11(7):e045469. doi: 10.1136/bmjopen-2020-045469 (PMC8318720; doi:10.1136/bmjopen-2020-045469)
Supplement: Author's manuscript [file bmjopen-2020-045469.draft_revisions.pdf]

BMJ Open

BMJ Open is committed to open peer review. As part of this commitment we make the peer review history of every article we publish publicly available.

When an article is published we post the peer reviewers' comments and the authors' responses online. We also post the versions of the paper that were used during peer review. These are the versions that the peer review comments apply to.

The versions of the paper that follow are the versions that were submitted during the peer review process. They are not the versions of record or the final published versions. They should not be cited or distributed as the published version of this manuscript.

BMJ Open is an open access journal and the full, final, typeset and author-corrected version of record of the manuscript is available on our site with no access controls, subscription charges or pay-per-view fees (http://bmjopen.bmj.com).

If you have any questions on BMJ Open's open peer review process please email info.bmjopen@bmj.com

BMJ Open

Remote assessment of care home residents during the COVID-19 pandemic – what is the role of the National Early Warning Score? A qualitative interview study

Journal:	*BMJ Open*
Manuscript ID	bmjopen-2020-045469
Article Type:	Original research
Date Submitted by the Author:	01-Oct-2020
Complete List of Authors:	Stocker, Rachel; Newcastle University, Institute of Health and Society<br>Russell, Sian; Newcastle University, Population Health Sciences Institute<br>Liddle, Jennifer; Newcastle University, Population Health Sciences Institute<br>Barker, Robert; Newcastle University, Population and Health Sciences Institute<br>Remmer, Adam; South Tyneside and Sunderland NHS Foundation Trust<br>Gray, Joanne; Northumbria University,<br>Hanratty, Barbara; Newcastle University, Population Health Sciences Institute<br>Adamson, Joy; University of York
Keywords:	COVID-19, GERIATRIC MEDICINE, Organisation of health services < HEALTH SERVICES ADMINISTRATION & MANAGEMENT, Quality in health care < HEALTH SERVICES ADMINISTRATION & MANAGEMENT, PRIMARY CARE, QUALITATIVE RESEARCH

SCHOLARONE™
Manuscripts

I, the Submitting Author has the right to grant and does grant on behalf of all authors of the Work (as defined in the below author licence), an exclusive licence and/or a non-exclusive licence for contributions from authors who are: i) UK Crown employees; ii) where BMJ has agreed a CC-BY licence shall apply, and/or iii) in accordance with the terms applicable for US Federal Government officers or employees acting as part of their official duties; on a worldwide, perpetual, irrevocable, royalty-free basis to BMJ Publishing Group Ltd ("BMJ") its licensees and where the relevant Journal is co-owned by BMJ to the co-owners of the Journal, to publish the Work in this journal and any other BMJ products and to exploit all rights, as set out in our licence.

The Submitting Author accepts and understands that any supply made under these terms is made by BMJ to the Submitting Author unless you are acting as an employee on behalf of your employer or a postgraduate student of an affiliated institution which is paying any applicable article publishing charge ("APC") for Open Access articles. Where the Submitting Author wishes to make the Work available on an Open Access basis (and intends to pay the relevant APC), the terms of reuse of such Open Access shall be governed by a Creative Commons licence – details of these licences and which Creative Commons licence will apply to this Work are set out in our licence referred to above.

Other than as permitted in any relevant BMJ Author's Self Archiving Policies, I confirm this Work has not been accepted for publication elsewhere, is not being considered for publication elsewhere and does not duplicate material already published. I confirm all authors consent to publication of this Work and authorise the granting of this licence.

For peer review only

Remote assessment of care home residents during the COVID-19 pandemic – what is the role of the National Early Warning Score? A qualitative interview study

Rachel Stocker[1,3], Siân Russell[1], Jennifer Liddle[1], Robert Oliver Barker[1], Adam Remmer[2], Joanne Gray, Barbara Hanratty[1], Joy Adamson[3]

1. Population Health Sciences Institute, Newcastle University
2. South Tyneside and Sunderland NHS Foundation Trust
3. Department of Health Sciences, University of York

Address correspondence to Dr Rachel Stocker, Population Health Sciences Institute, Newcastle University, Level 2, Newcastle Biomedical Research Building Campus for Ageing and Vitality, Newcastle upon Tyne NE4 5PL, UK.

Email: rachel.stocker@newcastle.ac.uk

Abstract

Background

The COVID-19 pandemic has taken a heavy toll on the care home sector, with residents accounting for up to half of all deaths in Europe. The response to acute illness in care homes plays a particularly important role in the care of residents during a pandemic. Digital recording of a National Early Warning Score (NEWS2), which involves the measurement of physical observations, started in one area of England in 2016. Implementation of NEWS was accelerated in the early stages of the pandemic, despite limited evidence for its use in the care home setting.

For peer review only - http://bmjopen.bmj.com/site/about/guidelines.xhtml

Objectives

To evaluate the use and perceived utility of the NEWS2 intervention in care homes in one area of North-East England during the COVID-19 pandemic, from the perspective of stakeholders involved in care delivery and commissioning.

Methods

A qualitative interview study with care home (n=10) and NHS (n=7) staff. Data were analysed using thematic analysis.

Results

Use of NEWS2 in care homes in this area accelerated during the COVID-19 pandemic. Stakeholders felt that NEWS2, and its education and support package, improved the response of care homes and healthcare professionals to deterioration in residents' health during the pandemic. NEWS2 components, particularly temperature and oxygen saturation, were also measured independently as potential indicators of COVID-19 disease. Healthcare professionals valued the ability to remotely monitor resident observations, which facilitated triage and treatment decisions. Care home staff felt empowered by NEWS2, providing a common clinical language to communicate concerns with external services, and acting as an adjunct to staff intuition of resident deterioration.

Conclusions

The NEWS2 intervention formed an important part of the care home response to COVID-19 in the study area. Positive staff perceptions now need to be supplemented with data on the impact on resident health and wellbeing, workload, and service utilisation, during the COVID-19 pandemic and beyond.

Strengths and limitations of this study

- This study is the first to examine the role of the National Early Warning Score (NEWS2) in care homes during the COVID-19 pandemic in the UK.
- Care home and NHS staff were recruited and interviewed at the height of the pandemic (May 2020), providing an important insight into the COVID-19 response in this sector of care.
- The role of NEWS2 in identifying COVID-19 disease, and monitoring resident health more generally, was explored in-depth with semi-structured remotely conducted interviews.

For peer review only - http://bmjopen.bmj.com/site/about/guidelines.xhtml

- General practitioners and care home residents were not recruited due to the pressures on the NHS and care home sector; future research should directly seek rather than infer their views.

For peer review only

For peer review only - http://bmjopen.bmj.com/site/about/guidelines.xhtml

Introduction

The care home sector is one of the most overlooked components of health and social care in the UK. Care homes provide accommodation, personal care and support, and companionship to some of the most vulnerable, frail, and medically complex older adults in society. There are more than 11,000 care homes in the UK[1], home to over 400,000 residents, representing 16% of those aged 85 or over. Care homes may receive some funding from the NHS: the majority of funding comes from local authorities, or from residents or residents' families.

Care homes have been severely affected by the COVID-19 pandemic. The virus spread rapidly and many residents died in a short space of time. Estimates suggest that up to half of all COVID-19 related deaths in Europe were care home residents[2], and people working in social care are dying at twice the rate of the general population.[3] This situation has been described by the World Health Organisation as an "unimaginable human tragedy[4]". Many care homes have struggled with staffing, accessing personal protective equipment (PPE)[5], and maintaining isolation measures during the pandemic.

In recent years, efforts have been made to support the care home sector to improve the quality of their health-related care.[6] Identifying and monitoring residents for early signs of acute illness, using tools such as the National Early Warning Score (NEWS2), has been a particular area of interest.[7] The NEWS2 was initially developed for use in hospital settings. It requires the measurement of six parameters: temperature, pulse, systolic blood pressure, respiratory rate, oxygen saturation and level of consciousness. The overall NEWS triggers a response, ranging from repeating the measurements within a specific time frame, to initiating an emergency medical response.

Implementation of the NEWS into care homes is already underway across multiple regions in the UK[8 9], despite a lack of validation and proven effectiveness outside the hospital setting.[10] The aim is to improve the response to acute illness by improving triage and communication with external healthcare professionals, and to reduce avoidable hospital admissions. However, concerns have been expressed about the appropriateness of using the NEWS in care homes, including potential adverse influences on palliative care.[11] NEWS2 was also designed for use by clinically trained staff, such as registered nurses, who are not on-site in residential care facilities. Previous work indicates that whilst the collection of NEWS2 in care homes appears to be feasible[12], the complexity of the care home context and subsequent intricacies in implementing NEWS2 need careful thought and planning to exploit any potential benefits for residents.[13 14]

For peer review only - http://bmjopen.bmj.com/site/about/guidelines.xhtml

Early in the COVID-19 pandemic, the British Geriatrics Society emphasised the importance of care homes having the skills, training, and equipment to identify deterioration in residents, and advocated the use of systems that incorporate NEWS2.[15] One Clinical Commissioning Group (CCG) in the North-East of England accelerated the roll out of NEWS2 in care homes, with the aim of ensuring all residential and nursing homes have the equipment necessary to measure residents' physical observations and calculate a NEWS. The NEWS2 intervention (equipment for taking vital signs and digital tablet for recording NEWS2) was implemented into care homes in this CCG in 2016. In 2019 the implementation process was revised and a nurse was appointed in an educator role to train care home staff and support care homes. This paper presents a qualitative interview study with key stakeholders, with the aim of understanding how NEWS2 has been used in care homes during the COVID-19 pandemic, and how it has influenced resident care and outcomes.

Methods

Semi-structured interviews were conducted with 17 stakeholders in a CCG area in the North-East of England. A phenomenological methodological approach was used to underpin the study. Stakeholders were identified using purposive sampling, through existing links with local NHS staff. Participants were also asked to identify other colleagues involved in the NEWS2 rollout (snowball sampling). We sought to include staff from a range of residential and nursing care homes, particularly with varied experience using NEWS2. A key NHS employee, known to potential participants, acted as a gatekeeper, sending introductory emails with a participant information sheet. Once consent to be contacted by the research team was secured, a researcher RS, made direct contact by email to invite participation. All individuals approached agreed to take part. Informed consent was secured either electronically or verbally in line with Health Research Authority principles. Table 1 describes our participant sample.

Table 1. Interviewees and their working role

Working role	Sector	n
**Care home manager**	Care home	7
**Care home senior carer/nurse**	Care home	3
**Director**	NHS	1
**Director**	NHS-related body	1

For peer review only - http://bmjopen.bmj.com/site/about/guidelines.xhtml

**Specialist nurse**	NHS	4
**Commissioning**	NHS	1
	**Total:**	**17**

All interviews were carried out remotely by telephone or videoconferencing, during May 2020. Development of an interview topic guide (Box 1) was informed by previous research conducted by the team, published literature evaluating the use of NEWS2 in care homes, and relevant literature and direct communications to the research team describing care home experiences of the pandemic. Field notes were taken after interviews.

Box 1. Interview topics

- Perceived validity of NEWS2 in care homes
- Experiences of using NEWS2 before, and during the pandemic
- NEWS2 training for care homes
- Perceptions on the usefulness of NEWS2 during the pandemic, including identification of potential cases
- How NEWS2 influenced decision making and triage, within and outwith care homes
- Variations in use of NEWS2 during the pandemic

Interviews were carried out by RS or SR (Both female Research Associates, doctorates in health services research and medical sociology, and experience of qualitative studies in care homes), audio recorded and transcribed verbatim. All transcripts were anonymised. Interviews lasted 30-60 minutes. Approval was granted by Newcastle University Faculty of Medical Sciences Research Ethics Committee (ref: 2569/2020) and NHS North of England Commissioning Support; HRA approvals were not needed as the work was deemed to be a service evaluation.

Patient and public involvement

Patients and the public were first involved in this study at the idea generation stage, via the Newcastle University Care Home Interest PPI Group (CHIG). The research questions, topic guide, and study design were discussed via email with group members before the study commenced. CHIG members stressed the importance of carrying out interviews remotely and flexibly to time constraints, given the burden of the pandemic on the health and social care sector. Our discussions with this PPI group confirmed that COVID-19 related research in care homes is viewed as a pressing research priority. PPI partners will be consulted on appropriate dissemination strategies.

For peer review only - http://bmjopen.bmj.com/site/about/guidelines.xhtml

Data analysis

RS and SR conducted a thematic analysis of the dataset, following the principles of Braun and Clark's six-phase framework[16]. NVivo 11 software was used to aid in data management. Transcripts were read in full, then coded line by line, separately, by RS and SR. Codes were compared within and between transcripts, using the constant comparative technique[17], to streamline the coding framework. Emergent themes were discussed within the study team, and through the use of the six-phase framework, linked together to form a final set of themes and sub-themes. Interviews ceased when no new themes were identified from the data (data saturation) as agreed by RS and SR.

Results

Analysis produced four higher level themes from the data: 1) NEWS2 pre-pandemic: embedded practically and attitudinally; 2) NEWS2 during the pandemic: an adjunct to COVID-19 identification; 3) Empowerment of care home staff with the NHS agenda and language; 4) Centrality of training relationships and clinical support for accelerated implementation of NEWS2 during a pandemic.

Theme 1. NEWS2 pre-pandemic: embedded practically and attitudinally

Care homes in this study were already familiar with NEWS2 before the COVID-19 pandemic and attitudes towards its use and usefulness were positive. Local NHS service commissioners believe that NEWS2 improves the response to acute unwell care home residents. They have invested in its implementation despite limited evidence for its effectiveness in this setting.

Training and supporting care homes in the use of the NEWS2 intervention

The education and support package for the NEWS2 intervention was delivered by an NHS clinical educator who had a nursing background. Training for NEWS2 for care homes in this area built on existing skills of staff, contextualising NEWS2 observations and theory with the importance of 'soft signs' of deterioration. This was compared positively to a previous model of training, which focused more on the practicalities of using the equipment to take observations and the computer tablet, delivered by a non-clinician.

For peer review only - http://bmjopen.bmj.com/site/about/guidelines.xhtml

I'm coming from a clinical perspective and [in the NEWS training and support] we'll do practicals, we'll go to the residents. […] And if for instance they scored low for the blood pressure, we could talk that through and we could identify possibilities why it would be low. [..] I think [care home staff] learn more that way. […] So it's less task orientated, and it made them step back a little bit, assess the situation and go, 'Right. I'm looking at the whole picture and I'm doing my NEWS score to kind of give me that reassurance,' that what they're seeing is correct. NHS specialist nurse 1

Integration and routinisation in everyday care home practice

Stakeholders described how the NEWS2 has become embedded and habitualised into the everyday routines of care homes and staff interactions with external healthcare services. Care homes recorded NEWS2 baseline scores regularly (for routine monitoring and comparison when residents became acutely unwell), except for those near the end of life. A NEWS2 was frequently requested by community healthcare professionals responding to concerns from care homes, and staff often calculated a NEWS before contacting healthcare professionals.

Perceived influence of NEWS in speeding up triage and treatment decisions

Care home and NHS healthcare professionals highlighted how NEWS2 could speed up initial triage and facilitate remote treatment decisions, expediting the process for residents to receive the care they required. Referrals accompanied by a NEWS2 score reduced the need for clinicians to visit care homes to take physical observations. Identification of early illness was considered to be more efficient with the use of NEWS2. Staff believed that illness was treated earlier, potentially avoiding unnecessary hospital admission.

[int: how would you say [using NEWS] has influenced the care of the residents in [area]?] I think they get a quicker response, a quicker care pathway […] Observations are a very powerful tool, particularly to GPs and they always like to know everybody's observations before they do any sort of clinical task or any decision I think. It probably changes the patient journey to get things quicker because the GPs have got the information that they are wanting to get without having to leave their room. Without having to go into the care home. NHS specialist nurse 2

For peer review only - http://bmjopen.bmj.com/site/about/guidelines.xhtml

For peer review only

Theme 2. NEWS2 during the pandemic: an adjunct to COVID-19 identification

At the outbreak of the COVID-19 pandemic in the UK and the start of lockdown measures, use of the NEWS2 by care homes in this area increased substantially. Use of NEWS2 with the specific intention of identifying possible COVID-19 infection varied across the care homes in our study. Stakeholders highlighted the power of the NEWS2 in identifying early illness due to COVID-19 as well as other causes, and the ability of the NEWS2 kit to facilitate remote monitoring thereby decreasing the need for healthcare professionals to visit care homes.

The use of individual physiological observations to identify COVID-19

If COVID-19 was suspected, care home staff frequently measured temperature and oxygen saturations to use as individual physiological measures, as opposed to the amalgamated NEWS. The use of these physiological observations for identifying COVID-19 disease was advocated by a local NHS network. Care home staff appreciated the importance of responding to isolated abnormalities of temperature or oxygen saturations to identify COVID-19, even if the overall NEWS was not triggering a high level of concern. Stakeholders remained broadly sceptical about the sensitivity and specificity of these clinical observations to accurately identify COVID-19 in this population – feeling that they went some way to help, and could prompt further investigation of potential COVID-19, but could not be conclusive.

> *[There are] two main symptoms with our experience, high temperature and low oxygen saturations. We've found in a couple of our residents, […] they had no temperature, and no breathing difficulty, but I couldn't understand - and when we checked their NEWS score we found oxygen saturation was low. So it helps us in the early diagnosis and to do appropriate support for the residents.* Care home manager 1

> *The response to the infection is hugely physiological. So NEWS is a way of prompting you to do physiology. […] I think if you'd asked me six months ago I'd have said 'Actually the NEWS score is more useful than the underlying physiology.' Actually, I'd be tempted to go the other way around now and say 'You do a NEWS score because it makes you look at the physiology and you're going to go, oh my god, look at that oxygen saturation.'* NHS director

For peer review only - http://bmjopen.bmj.com/site/about/guidelines.xhtml

Frequency of NEWS2 recordings and individual observations significantly increased month-on-month from March 2020 onwards, with some care homes taking baseline NEWS2s several times a week. Other care homes recorded the NEWS2 sporadically, suggesting wide variation in the use of NEWS2 in this area during the pandemic. Figure 1 illustrates the increase in NEWS2 and individual score recording before and during the pandemic.

FIGURE 1 HERE

Using NEWS2 and associated equipment to remotely monitor health

Another positive aspect of the NEWS2 intervention during the pandemic was to facilitate remote monitoring of residents' health by healthcare professionals. This reduced unnecessary footfall and infection risk, which was a major driver to the accelerated roll out of the NEWS2 intervention during the pandemic.

> *We had to just take a stand and reflect and say, 'What do we need to do? We know that the predictions [are that COVID-19] is going to come. We're likely to have to go into lockdown. Nobody is going to be able to go into care homes.' So we thought, 'actually NEWS is an important tool in that. […] We just need to respond'* NHS commissioner

> *CCGs have been formally told to set up remote MDT care home rounds, so they are having to put the IT in place. […] You need a digital way of recording [resident] observations, and then you can put in a safety netting process that will allow the care home to get back in touch. Because there's going to be loads of [residents] where you're going to go, 'they're not quite right but I definitely don't want to send them to hospital.'* NHS director

The ability for NHS staff, such as community nurses, to remotely access physiological observations and compare with baselines, was of particular value. The photograph facility of the NEWS2 tablet was also unlocked in response to the pandemic, giving external healthcare professionals the ability to assess and treat wounds remotely.

For peer review only - http://bmjopen.bmj.com/site/about/guidelines.xhtml

[int: Are there any doctors or GPs who are taking it upon themselves to log on to the [cloud system] and look at the NEW scores?] Yes, absolutely. [Pre-pandemic] I don't think it was utilised [by them], especially not as much as it could have been, but due to the COVID crisis, everyone across the city now want access to it and it's obviously taken a pandemic to realise how valuable the tool is. NHS specialist nurse 1

I think the biggest change for the [use of NEWS2 package] at the moment is the pictures. […] For example, if there was a patient who had a skin flap […] We'll say, 'Will you take a photo? Will you upload it so we can see it?' We can physically see that photo and then we can assess the severity, we can assess when the visit should next take place […] it's brilliant for that aspect. NHS specialist nurse 2

Theme 3: Empowerment of care home staff with the NHS agenda and language

Using the NEWS2 system to help identify and triage responses to deteriorating resident health was described by stakeholders as empowering. The NEWS2 package represented 'hard clinical data' which care home staff could generally rely upon to back up their intuition that the resident was unwell (termed 'soft signs' e.g. decreased appetite). Care home staff felt confident that with a NEWS2 reading, their concerns about deteriorating residents would be taken seriously by external healthcare professionals, as it evidenced their concerns. This was compared positively to care home staff's previous experiences seeking help from primary care, where staff often felt that concerns were not listened to. This feeling of empowerment was capitalised upon by both care homes and local NHS services during the COVID-19 pandemic.

When we've rang the likes of home recovery or 111 and we're challenged as to why we're ringing, when we can't really put a finger on it that somebody's not well. Now, this [NEWS] has really aided us to be able to look for those signs, look for those symptoms, using the NEWS score and being able to give better communication across the telephone as to why and what has happened with that particular resident. I know there's been times where we've kind of been brushed off by services. And I'm a big believer in our care staff, they may not be qualified nurses but they know their residents sometimes better than we do. […] Now I feel like we've got this tool now to be able to kind of prove that somewhat as well, and it does help back up what we're saying when it comes to our residents. So it's been great. Care home manager 2

For peer review only - http://bmjopen.bmj.com/site/about/guidelines.xhtml

During the COVID-19 pandemic, care home staff were relied upon by local NHS colleagues more than ever before to take on clinical procedures and observations traditionally carried out by visiting NHS healthcare professionals such as GPs and nurses. This challenge to traditional role boundaries was welcomed and encouraged by NHS stakeholders, as it met the needs of the NHS to reduce hospital admissions and footfall into care homes. In the early days of the pandemic and the absence of straightforward guidance for care homes, care home staff also welcomed this extension to their roles with NEWS2 use, as they could measure and focus on clinical observations – in other words, the NEWS2 package facilitated a more concrete response within care homes to tackling a then-unknown virus.

> *[int: What do you think about the use of NEWS2 during this pandemic in care homes?] Well I've always loved it. I really love the NEWS tablet and for why, because I think it totally empowers the care staff. […] Care staff, when they say, 'They're just not right. I don't know what's wrong with them, but the person's not right.' Now they've got a little more ammunition to say, 'well actually, such-and-such isn't very well. These are the observations…' and it makes them have more of a voice. That's what I love about it so when they ring the GP and they say, 'Well actually the observations are blah, blah, blah,' and they're almost listened to a little bit more because they've got some sort of clinical information rather than just saying, 'Oh I don't know what's wrong with them.' I think that applies to here and now as well with the pandemic.*
> NHS specialist nurse 2

Theme 4. Centrality of training relationships and clinical support for accelerated implementation of NEWS2 during a pandemic

When the pandemic broke out, the CCG commenced a rapid roll out of the NEWS2 intervention to the minority of care homes in the area that were waiting for implementation. Care homes already using the NEWS2 package before the pandemic were contacted by the clinical educator to reinforce that support was available. The pandemic highlighted the importance of care homes, particularly those without registered nursing staff, having an engaged and supportive nominated NHS clinical educator.

For peer review only - http://bmjopen.bmj.com/site/about/guidelines.xhtml

Training and implementing the NEWS2 package in care homes during the pandemic

Training to support rapid implementation during the pandemic consisted of a short, socially distanced visit from the clinical educator to the care home, training one or two key members of staff in the home rather than a larger body of staff, and without practical examples of training. This rapid roll out model demonstrated that relationships built during training were integral to ongoing NEWS2 use and understanding. The training constraints related to the pandemic were perceived as having had a major impact on the quality of the rapid implementation and confidence of staff.

> *[During the rapid training], I don't feel like I built great rapport. I don't feel like it instilled that much confidence in them and I haven't really received many phone calls from those homes. Yes they're doing NEWS, but usually the homes that I've really been visibly in, really supported them and built those rapports and stuff up with, I feel like I get more phone calls which is great because they want support which then that in turn means they've picked up something, they're looking for answers and help and they get it. Where those other homes, I would worry that maybe they don't feel as confident to pick up the phone and speak with me because I've raced in, did what I had to do and then left – and it's very difficult to build up rapport over time, and trust.* NHS specialist nurse 1

Ongoing clinical support for care homes during the pandemic

Emphasis was placed on the importance of ongoing clinical support for care homes. In this area, care homes had access to the clinical educator and a specialist nurse with a specific care home remit. Having these key contacts, who were knowledgeable and approachable, was valued by care homes and capitalised upon during the COVID-19 pandemic. They were able to build upon long-term relationships with each other, cultivating understanding of each sector and respect for each other's role and remit. This cross-sector team working helped to legitimise use of NEWS2 in a non-NHS setting, and smoothed many processes of integrated care over traditional NHS and social care boundaries.

> *This is not just about the technical or the medical aspect, it's often about the relationships that [clinical educator] is then building up. […] I think that methodology has worked because you're consolidating it with a really good grip and really getting [care homes] to be the expert.* NHS commissioner

For peer review only - http://bmjopen.bmj.com/site/about/guidelines.xhtml

[Clinical educator] is a very approachable person. You can email him, you phone him and in all fairness, we would probably see [clinical educator] at least probably once a week. He'll pop in just to make sure everything is alright and we're well, you know, and if we're having any problems. So that support is continually there. Care home manager 4

Discussion

Summary of findings

During the COVID-19 pandemic, the use of NEWS2 in care homes in this area became increasingly embedded into daily care home and NHS practices, and was viewed as a key aspect of the pandemic response in this setting. Care home staff, NHS healthcare professionals and service commissioners shared a belief that measuring NEWS, its component physiological observations, alongside the associated education and support package, have enhanced the response to deteriorating resident health during the pandemic.

It is important to highlight that many of the benefits espoused of the NEWS2 intervention relate more to the training and support package, and the equipment, rather than the NEWS2 itself. This particular NEWS2 intervention permits the measurement of physiological observations, which are transferred by Bluetooth to the digital tablet and a NEWS is automatically generated. The observations and NEWS are available to external healthcare professionals on cloud storage, with permits remote monitoring. This has been particularly helpful during the pandemic, reducing footfall into care homes, but is not necessarily a benefit stemming directly from the NEWS2 overall scoring system. Similarly, the training and support package offered by the local NHS nursing support services also focuses on identifying 'soft signs' of deterioration alongside the objective measure in the NEWS2. One key aspect of the NEWS2 package success in care homes in this area, made even more apparent during the pandemic, is the clinical educator who was an NHS clinician with experience and understanding of the needs of the care home sector. The NEWS2 package is a complex intervention, making it difficult to 'unpack the blackbox of improvement'[18] and decipher the degree to which the different components of the intervention contributed to the perceived benefit.

A prominent finding was how the NEWS2 intervention empowered care home staff. For care homes, the NEWS2 package represented a helpful link to the NHS. Having a common clinical language which

For peer review only - http://bmjopen.bmj.com/site/about/guidelines.xhtml

care home staff could use – or leverage for action, where necessary – was a useful tool to promote more integrated working between care homes and the NHS. It also allowed for quicker initial triage to appropriate services, often negating the need for a primary care healthcare professional to physically attend, which is an important consideration during the pandemic.

Comparison with other work

Previous evaluations of the use of NEWS2 in a non-hospital setting reflect many of our findings, including the benefits of using a common clinical language, and facilitating communication between sectors.[19] The importance of using NEWS2 as a part of a wider assessment of health, rather than a ritualistic, task-oriented procedure[20], has been stressed by both the UK Royal College of Physicians[7] and those evaluating its use in other settings including primary[11] and community care.[19] The clinician-led training and ongoing support model for the NEWS2 package in place in this area addresses these concerns.

There is some evidence to support the use of NEWS2 in the identification of and triage of hospital patients with suspected COVID-19.[21 22] However, there have been calls for NEWS2 to be modified with a more sensitive score for oxygen demand to better account for the development of hypoxic respiratory failure in COVID-19 patients.[23] Evidence for the use of NEWS2 in community settings during the pandemic remains sparse. There is little comparable evidence available to evaluate the utility of NEWS2 in care homes specifically during the COVID-19 pandemic. Some emerging evidence suggests that NEWS may have a role in population level disease surveillance in care homes, and highlights the potential utility of individual oxygen saturation, respiratory rate, and temperature measurements.[24] Early research findings also indicate that up to half of care home residents are asymptomatic when they test positive for COVID-19[25], reflecting the experiences of interviewees in this study.

Implementing complex health interventions in the care home sector can be difficult, and requires care home engagement and support, and an understanding of their needs.[13 26] Having an NHS clinical educator, who was experienced in care home work, played a significant part in the NEWS2 package perceived success. Ongoing support to care homes legitimised and enhanced cross-sector working, which are important factors in effective healthcare delivery to this important sector of care.[27]

For peer review only - http://bmjopen.bmj.com/site/about/guidelines.xhtml

Strengths and limitations

We were able to interview ten care home staff from seven care homes in the locality, despite the pandemic related pressure on this sector during the interviewing period (May 2020). This represents a strength of our study and highlights the importance that interviewees placed on the topic and expressing their views. However, we were unable to interview GPs due to the pressure the pandemic has placed on primary care. The pandemic visiting restrictions in care homes also meant that we were unable to interview any care home residents themselves. Future work should include recruitment of care home residents, and GPs, so that their views are directly included rather than inferred.

Conclusion

Use of NEWS2 in care homes in this CCG area was viewed as business as usual. Uptake and use accelerated significantly at the start of the COVID-19 pandemic, without an evidence base indicating its usefulness in such a situation. In the initial absence of comprehensive government guidance for care homes, NEWS2 was relied upon to meet the needs of both care homes and the NHS response to the pandemic, particularly as an adjunct to identifying COVID-19 and remotely monitoring deteriorating resident health. It is important to separate out the role of the training, support and equipment, to the NEWS2 itself, when evaluating the efficacy of the intervention. This model is specific to the local area and service set up, which highlights the difficulty of evaluating similar cross-sector complex interventions in other geographical areas. The perceived success of the NEWS2 intervention in this CCG may point to the usefulness of the NEWS2 vital observation and cloud storage package itself during the pandemic, rather than the NEWS2 theory itself. However, the empowering nature of the NEWS2 package more generally, and the ongoing training and support to care homes provided by a key link NHS educator, are important aspects of the perceived success of NEWS2 in care homes in this area. There is very little published evidence on the use of NEWS in care homes, despite growing rates of use. Future studies could include consensus work about best practice of NEWS use in this important sector of care, and desired outcomes.

Contributors

RS, SR and JA designed the study with JL and BH. RS and SR conducted interviews. RS, SR, and JA analysed data. RS, SR and JA drafted the article, and AR, JL, JG and BH performed critical revision of the article for important intellectual content. RS is guarantor of the paper. All authors approved the final version to be published.

For peer review only - http://bmjopen.bmj.com/site/about/guidelines.xhtml

Data sharing statement

Data are not publicly available.

Word count

4716 words.

Declaration of Conflicts of Interest

The authors declare no conflicts of interest.

Declaration of Funding

This study is funded by the National Institute for Health Research (NIHR), Research for Patient Benefit [award number PB-PG-0418-20034]. The views expressed are those of the author(s) and not necessarily those of the NIHR or the Department of Health and Social Care.

References

1. Competition and Markets Authority. Care home market study summary of final report. London, 2017.
2. Office for National Statistics. Deaths registered weekly in England and Wales, provisional 2020 [Available from: https://www.ons.gov.uk/peoplepopulationandcommunity/birthsdeathsandmarriages/deaths/datasets/weeklyprovisionalfiguresondeathsregisteredinenglandandwales.
3. Office for National Statistics. Coronavirus (COVID-19) related deaths by occupation, England and Wales: deaths registered up to and including 20 April 2020 2020 [Available from: https://www.ons.gov.uk/releases/covid19relateddeathsbyoccupationenglandandwalesdeathsregistereduptoandincluding20thapril2020.
4. World Health Organization. Statement – Invest in the overlooked and unsung: build sustainable people-centred long-term care in the wake of COVID-19 2020 [Available from: http://www.euro.who.int/en/media-centre/sections/statements/2020/statement-invest-in-the-overlooked-and-unsung-build-sustainable-people-centred-long-term-care-in-the-wake-of-covid-19.
5. Brainard J, Rushton S, Winters T, et al. Introduction to and spread of COVID-19 in care homes in Norfolk, UK. [Preprint, available at https://wwwmedrxivorg/content/101101/2020061720133629v1fullpdf] 2020.
6. NHS England. NHS Five Year Forward View. London, 2014.
7. Royal College of Physicians. National Early Warning Score (NEWS) 2: Standardising the assessment of acute-illness severity in the NHS. Updated report of a working party. London: RCP, 2017.
8. West Hampshire Clinical Commissioning Group. RESTORE2: recognise early soft-signs, take observations, respond, escalate 2019 [Available from: https://www.westhampshireccg.nhs.uk/restore2-training-and-resources.

For peer review only - http://bmjopen.bmj.com/site/about/guidelines.xhtml

9. Hodgson P, Cook G, Thompson J, et al. Assessment and clinical decision making of the acutely ill older care home resident: implementation of NEWS in Gateshead Care Homes. Report., 2017.
10. Finnikin S, Hayward G, Wilson F, et al. Are referrals to hospital from out-of-hours primary care associated with National Early Warning Scores? Emergency Medicine Journal 2020;**37**(5):279-85.
11. Greenhalgh T, Treadwell J, Burrow R, et al. Should we use the NEWS (or NEWS2) score when assessing patients with possible COVID-19 in primary care?: Oxford COVID-19 Evidence Service, 2020.
12. Barker RO, Stocker R, Russell S, et al. Distribution of the National Early Warning Score (NEWS) in care home residents. Age and Ageing 2019;**49**(1):141-45.
13. Russell S, Stocker R, Barker RO, et al. Good NEWS for care homes? A qualitative evaluation of the implementation of the National Early Warning Score in UK care homes. British Journal of General Practice 2020;**In press**.
14. Hodge S, Thompson C, Gordon AL. National early warning scores in care homes: do policy imperatives reflect a genuine need? Age and Ageing 2019;**49**(1):5-6.
15. British Geriatrics Society. COVID-19: Managing the COVID-19 pandemic in care homes for older people. Good Practice Guide 2020 [Available from: https://www.bgs.org.uk/resources/covid-19-managing-the-covid-19-pandemic-in-care-homes#anchor-nav-identifying-residents-who-may-have-covid-19-and-how-to-respond.
16. Braun V, Clarke V. Using thematic analysis in psychology. Qualitative research in psychology 2006;**3**(2):77-101.
17. Glaser BG. The constant comparative method of qualitative analysis. Social problems 1965;**12**(4):436-45.
18. Ramaswamy R, Reed J, Livesley N, et al. Unpacking the black box of improvement. International Journal for Quality in Health Care 2018;**30**(suppl_1):15-19.
19. Brangan E, Banks J, Brant H, et al. Using the National Early Warning Score (NEWS) outside acute hospital settings: a qualitative study of staff experiences in the West of England. BMJ Open 2018;**8**(10):e022528.
20. Massey D, Chaboyer W, Anderson V. What factors influence ward nurses' recognition of and response to patient deterioration? An integrative review of the literature. Nursing Open 2017;**4**(1):6-23.
21. Myrstad M, Ihle-Hansen H, Tveita AA, et al. National Early Warning Score 2 (NEWS2) on admission predicts severe disease and in-hospital mortality from Covid-19 – a prospective cohort study. Scandinavian Journal of Trauma, Resuscitation and Emergency Medicine 2020;**28**(1):66.
22. Gidari A, De Socio GV, Sabbatini S, et al. Predictive value of National Early Warning Score 2 (NEWS2) for intensive care unit admission in patients with SARS-CoV-2 infection. Infectious Diseases 2020;**52**(10):698-704.
23. Lim NT, Pan D, Barker J. NEWS2 system requires modification to identify deteriorating patients with COVID-19. Clinical Medicine 2020;**20**(4):e133-e34.
24. Stow D, Barker RO, Matthews FE, et al. National Early Warning Scores (NEWS / NEWS2) and COVID-19 deaths in care homes: a longitudinal ecological study. medRxiv 2020:2020.06.15.20131516.
25. Graham NSN, Junghans C, Downes R, et al. SARS-CoV-2 infection, clinical features and outcome of COVID-19 in United Kingdom nursing homes. Journal of Infection 2020.
26. Stocker R, Bamford C, Brittain K, et al. Care home services at the vanguard: a qualitative study exploring stakeholder views on the development and evaluation of novel, integrated approaches to enhancing healthcare in care homes. BMJ Open 2018;**8**(3):e017419.

For peer review only - http://bmjopen.bmj.com/site/about/guidelines.xhtml

27. Gordon AL, Goodman C, Davies SL, et al. Optimal healthcare delivery to care homes in the UK: a realist evaluation of what supports effective working to improve healthcare outcomes. Age and Ageing 2018;**47**(4):595-603.

For peer review only

For peer review only - http://bmjopen.bmj.com/site/about/guidelines.xhtml

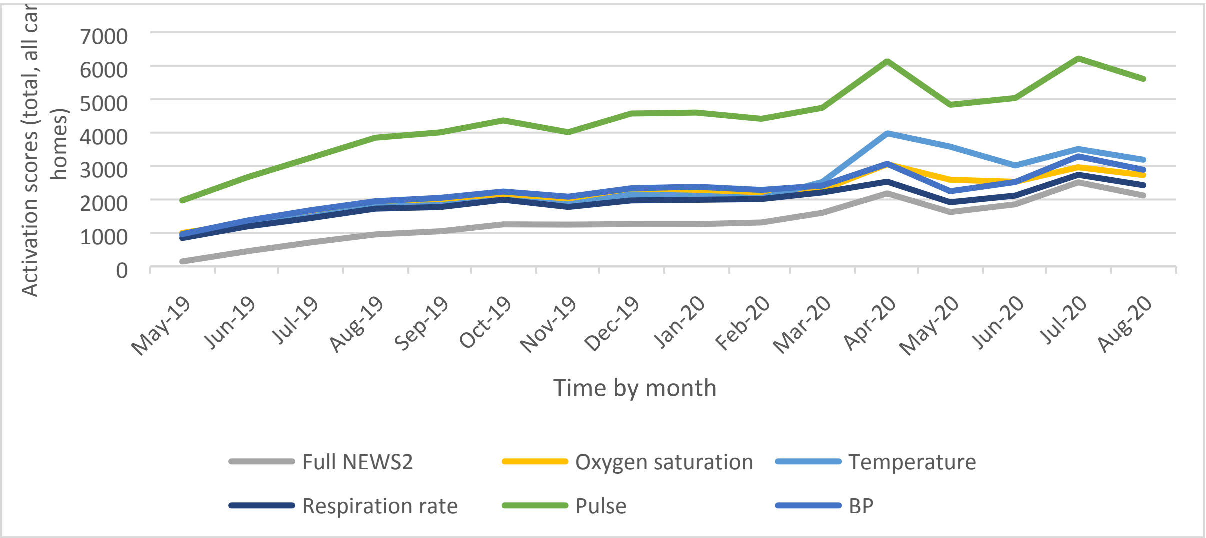

Figure 1 Frequency of NEWS2 and its component measures taken in care homes in the study area

For peer review only

Manuscript: **Remote assessment of care home residents during the COVID-19 pandemic – what is the role of the National Early Warning Score? A qualitative interview study**

Consolidated criteria for reporting qualitative studies (COREQ): 32-item checklist

Developed from:
Tong A, Sainsbury P, Craig J. Consolidated criteria for reporting qualitative research (COREQ): a 32-item checklist for interviews and focus groups. *International Journal for Quality in Health Care*. 2007. Volume 19, Number 6: pp. 349 – 357

No. Item	Guide questions/description	Reported on Page #
**Domain 1: Research team and reflexivity**		
Personal Characteristics		
1. Inter viewer/facilitator	Which author/s conducted the inter view or focus group?	RS and SR, p5
2. Credentials	What were the researcher's credentials? E.g. PhD, MD	PhDs, p5
3. Occupation	What was their occupation at the time of the study?	Research Associates, p5
4. Gender	Was the researcher male or female?	Both female, p5
5. Experience and training	What experience or training did the researcher have?	PhDs and significant experience in care home related qualitative research. P5
Relationship with participants		
6. Relationship established	Was a relationship established prior to study commencement?	No
7. Participant knowledge of the interviewer	What did the participants know about the researcher? e.g. personal goals, reasons for doing the research	Nothing other than the study information
8. Interviewer characteristics	What characteristics were reported about the inter viewer/facilitator? e.g. Bias, assumptions, reasons and interests in the research topic	None relevant

For peer review only

**Domain 2: study design**		
Theoretical framework		
9. Methodological orientation and Theory	What methodological orientation was stated to underpin the study? e.g. grounded theory, discourse analysis, ethnography, phenomenology, content analysis	Phenomenology, p4
Participant selection		
10. Sampling	How were participants selected? e.g. purposive, convenience, consecutive, snowball	Purposive and snowball sampling, p4
11. Method of approach	How were participants approached? e.g. face-to-face, telephone, mail, email	Email, p4
12. Sample size	How many participants were in the study?	17, p5
13. Non-participation	How many people refused to participate or dropped out? Reasons?	No refusals or drop outs, p4
Setting		
14. Setting of data collection	Where was the data collected? e.g. home, clinic, workplace	Home and workplace, p4-5
15. Presence of non-participants	Was anyone else present besides the participants and researchers?	No, p4
16. Description of sample	What are the important characteristics of the sample? e.g. demographic data, date	Information and table on p4-5
Data collection		
17. Interview guide	Were questions, prompts, guides provided by the authors? Was it pilot tested?	Yes, p5
18. Repeat interviews	Were repeat inter views carried out? If yes, how many?	No
19. Audio/visual recording	Did the research use audio or visual recording to collect the data?	Audio recording, p5
20. Field notes	Were field notes made during and/or after the inter view or focus group?	Afterwards, p5
21. Duration	What was the duration of the inter views or focus group?	30-60 minutes, p5
22. Data saturation	Was data saturation discussed?	Yes, p6
23. Transcripts returned	Were transcripts returned to participants for comment and/or correction?	No
**Domain 3: analysis and findings**		
Data analysis		
24. Number of data coders	How many data coders coded the data?	2, p6

For peer review only

25. Description of the coding tree	Did authors provide a description of the coding tree?	No (only themes and sub-themes, p6 onwards)
26. Derivation of themes	Were themes identified in advance or derived from the data?	Derived from the data, p6
27. Software	What software, if applicable, was used to manage the data?	NVivo, p5
28. Participant checking	Did participants provide feedback on the findings?	No
Reporting		
29. Quotations presented	Were participant quotations presented to illustrate the themes/findings? Was each quotation identified? e.g. participant number	Yes, p6 onwards
30. Data and findings consistent	Was there consistency between the data presented and the findings?	Yes
31. Clarity of major themes	Were major themes clearly presented in the findings?	Yes
32. Clarity of minor themes	Is there a description of diverse cases or discussion of minor themes?	Yes

For peer review only

BMJ Open

Experiences of a National Early Warning Score (NEWS) intervention in care homes during the COVID-19 pandemic: A qualitative interview study

Journal:	*BMJ Open*
Manuscript ID	bmjopen-2020-045469.R1
Article Type:	Original research
Date Submitted by the Author:	04-Mar-2021
Complete List of Authors:	Stocker, Rachel; Newcastle University, Institute of Health and Society<br>Russell, Sian; Newcastle University, Population Health Sciences Institute<br>Liddle, Jennifer; Newcastle University, Population Health Sciences Institute<br>Barker, Robert; Newcastle University, Population and Health Sciences Institute<br>Remmer, Adam; South Tyneside and Sunderland NHS Foundation Trust<br>Gray, Joanne; Northumbria University,<br>Hanratty, Barbara; Newcastle University, Population Health Sciences Institute<br>Adamson, Joy; University of York
**Primary Subject Heading**:	Geriatric medicine
Secondary Subject Heading:	Communication, General practice / Family practice, Health services research, Public health, Qualitative research
Keywords:	COVID-19, GERIATRIC MEDICINE, Organisation of health services < HEALTH SERVICES ADMINISTRATION & MANAGEMENT, Quality in health care < HEALTH SERVICES ADMINISTRATION & MANAGEMENT, PRIMARY CARE, QUALITATIVE RESEARCH

SCHOLARONE™
Manuscripts

I, the Submitting Author has the right to grant and does grant on behalf of all authors of the Work (as defined in the below author licence), an exclusive licence and/or a non-exclusive licence for contributions from authors who are: i) UK Crown employees; ii) where BMJ has agreed a CC-BY licence shall apply, and/or iii) in accordance with the terms applicable for US Federal Government officers or employees acting as part of their official duties; on a worldwide, perpetual, irrevocable, royalty-free basis to BMJ Publishing Group Ltd ("BMJ") its licensees and where the relevant Journal is co-owned by BMJ to the co-owners of the Journal, to publish the Work in this journal and any other BMJ products and to exploit all rights, as set out in our licence.

The Submitting Author accepts and understands that any supply made under these terms is made by BMJ to the Submitting Author unless you are acting as an employee on behalf of your employer or a postgraduate student of an affiliated institution which is paying any applicable article publishing charge ("APC") for Open Access articles. Where the Submitting Author wishes to make the Work available on an Open Access basis (and intends to pay the relevant APC), the terms of reuse of such Open Access shall be governed by a Creative Commons licence – details of these licences and which Creative Commons licence will apply to this Work are set out in our licence referred to above.

Other than as permitted in any relevant BMJ Author's Self Archiving Policies, I confirm this Work has not been accepted for publication elsewhere, is not being considered for publication elsewhere and does not duplicate material already published. I confirm all authors consent to publication of this Work and authorise the granting of this licence.

For peer review only

Experiences of a National Early Warning Score (NEWS) intervention in care homes during the COVID-19 pandemic: A qualitative interview study

Rachel Stocker[1,3], Siân Russell[1], Jennifer Liddle[1], Robert Oliver Barker[1], Adam Remmer[2], Joanne Gray, Barbara Hanratty[1], Joy Adamson[3]

1. Population Health Sciences Institute, Newcastle University
2. South Tyneside and Sunderland NHS Foundation Trust
3. Department of Health Sciences, University of York

Address correspondence to Dr Rachel Stocker, Population Health Sciences Institute, Newcastle University, Level 2, Newcastle Biomedical Research Building Campus for Ageing and Vitality, Newcastle upon Tyne NE4 5PL, UK.

Email: rachel.stocker@newcastle.ac.uk

For peer review only

Abstract

Background

The COVID-19 pandemic has taken a heavy toll on the care home sector, with residents accounting for up to half of all deaths in Europe. The response to acute illness in care homes plays a particularly important role in the care of residents during a pandemic. Digital recording of a National Early Warning Score (NEWS), which involves the measurement of physical observations, started in care homes in one area of England in 2016. Implementation of a NEWS intervention (including equipment, training and support) was accelerated early in the pandemic, despite limited evidence for its use in the care home setting.

For peer review only - http://bmjopen.bmj.com/site/about/guidelines.xhtml

Objectives

To understand how a National Early Warning Score (NEWS) intervention has been used in care homes in one area of North-East England during the COVID-19 pandemic, and how it has influenced resident care, from the perspective of stakeholders involved in care delivery and commissioning.

Methods

A qualitative interview study with care home (n=10) and NHS (n=7) staff. Data were analysed using thematic analysis.

Results

Use of the NEWS intervention in care homes in this area accelerated during the COVID-19 pandemic. Stakeholders felt that NEWS, and its associated education and support package, improved the response of care homes and healthcare professionals to deterioration in residents' health during the pandemic. Healthcare professionals valued the ability to remotely monitor resident observations, which facilitated triage and treatment decisions. Care home staff felt empowered by NEWS, providing a common clinical language to communicate concerns with external services, acting as an adjunct to staff intuition of resident deterioration.

Conclusions

The NEWS intervention formed an important part of the care home response to COVID-19 in the study area. Positive staff perceptions now need to be supplemented with data on the impact on resident health and wellbeing, workload, and service utilisation, during the pandemic and beyond.

Strengths and limitations of this study

- This study is the first to examine the role of a National Early Warning Score (NEWS) intervention in care homes during the COVID-19 pandemic in the UK.
- Care home and NHS staff were recruited and interviewed at the height of the pandemic (May 2020), providing an important insight into the COVID-19 response in this sector.
- The role of NEWS in identifying COVID-19 disease and monitoring resident health, was explored in-depth with semi-structured remotely conducted interviews.
- General practitioners and care home residents were not recruited for practical reasons; future research should directly seek rather than infer their views.

For peer review only - http://bmjopen.bmj.com/site/about/guidelines.xhtml

Introduction

The care home sector is one of the most overlooked components of health and social care in the UK. Care homes provide accommodation, personal care and support, and companionship to some of the most vulnerable, frail, and medically complex older adults in society. There are more than 11,000 care homes (nursing and non-nursing) in the UK[1], home to over 400,000 residents, representing 16% of those aged 85 or over. The sector is dominated by private, for-profit, providers, who provide around four-fifths of care home beds in the UK.[2] Care homes may receive some funding from the NHS, however the majority of funding comes from local authorities, or from residents or residents' families. Care home residents receive medical care from visiting National Health Service (NHS) primary care professionals, including general practitioners and community nurses. Care configuration of NHS services for care home residents varies significantly across the country. Some areas provide a varied multidisciplinary healthcare team to support care home residents; others focus on specialist nurse provision; others have no special service.[3]

Care homes have been severely affected by the COVID-19 pandemic. The virus spread rapidly and many residents died in a short space of time. Estimates suggest that up to half of all COVID-19 related deaths in Europe were care home residents[4], and people working in social care are dying at twice the rate of the general population.[5] This situation has been described by the World Health Organisation as an "unimaginable human tragedy[6]". Many care homes have struggled with staffing, accessing personal protective equipment (PPE)[7], and maintaining isolation measures during the pandemic.

In recent years, efforts have been made to support the care home sector to improve the quality of health-related care.[8] Identifying and monitoring residents for early signs of acute illness, using tools such as the National Early Warning Score (NEWS; the latest version of the scoring system is the 'NEWS2'), has been a particular area of interest.[9] The NEWS was initially developed for use in hospital settings. It requires the measurement of six parameters: temperature, pulse, systolic blood pressure, respiratory rate, oxygen saturation and level of consciousness. The overall NEWS triggers a response, ranging from repeating the measurements within a specific time frame, to initiating an emergency medical response. The NEWS is intended to serve as an adjunct to, as opposed to replacing, a clinical decision.[9]

Implementation of the NEWS into care homes is already underway across multiple regions in the UK[10 11], despite a lack of validation and proven effectiveness outside the hospital setting.[12] The aim is to improve the response to acute illness in care home residents by improving triage and communication with external healthcare professionals, and to reduce avoidable hospital admissions. In care homes, the NEWS intervention has generally been implemented as a 'package', which includes as a minimum

For peer review only - http://bmjopen.bmj.com/site/about/guidelines.xhtml

the training and equipment to measure the vital observations needed to generate the NEWS score itself. Some implementations of a NEWS package have additionally included an ongoing support programme to care homes, and the use of a cloud-based storage system to store (and sometimes transmit to primary care staff for use in triage) NEWS scores. Concerns have been expressed about the appropriateness of using the NEWS in care homes, including potential adverse influences on palliative care.[13] NEWS was also designed for use by clinically trained staff, such as registered nurses, who are not on-site in residential care facilities. Previous work indicates that whilst the collection of NEWS in care homes appears to be feasible[14], the complexity of the care home context and subsequent intricacies in implementing NEWS need careful thought and planning to exploit any potential benefits for residents.[15 16] Early in the COVID-19 pandemic, the British Geriatrics Society emphasised the importance of care homes having the skills, training, and equipment to identify deterioration in residents, and advocated the use of systems that incorporate NEWS.[17]

This study focuses on the ways in which one Clinical Commissioning Group (CCG) with a high level of socioeconomic deprivation in the North-East of England, utilised their NEWS intervention in response to the COVID-19 pandemic. Having initially implemented a NEWS intervention in 2016, providing care homes with the equipment for taking vital signs, a digital tablet for recording NEWS and associated training, in 2019 the CCG invested further when a nurse was appointed as a clinical educator to provide more tailored training and support to care home staff (see Box 1 for an outline of the NEWS intervention in this area). Whilst most care homes in the CCG had received the 2019 intervention, for those who had not the CCG accelerated the roll out of this, with the aim of ensuring all residential and nursing homes had the equipment and training necessary to measure residents' physical observations and calculate a NEWS.

Box 1: NEWS intervention

Pre-COVID-19

- Equipment for taking vital signs and digital tablet for measuring and recording NEWS.
- Education and support delivered by an NHS clinical educator with a nursing background covering: practical use of equipment, interpretation of individual vital signs and resulting NEWS scores, contextualising NEWS observations and underpinning scoring system with the importance of 'soft signs' of deterioration.
- Internet web-based cloud storage system, whereby NEWS scores are automatically uploaded from the tablet to the storage system. The NHS clinical educator and other NHS staff involved in care for each care home can access the web-based system to view NEWS scores.

Additional function in response to COVID-19

- Additional functionality offered by the equipment was unlocked thereby expanding the NEWS package in this area further - notably a picture-taking facility to upload pictures of wounds.

For peer review only - http://bmjopen.bmj.com/site/about/guidelines.xhtml

This paper presents a qualitative interview study with key stakeholders, with the aim of understanding how a National Early Warning Score (NEWS) intervention has been used in care homes during the COVID-19 pandemic, and how it has influenced resident care. Specific research questions were:

1. How have care home staff used the NEWS intervention to assess resident illness during the COVID-19 pandemic?
2. What is the experience of care home staff of using the NEWS intervention during the COVID-19 pandemic?
3. How has the COVID-19 pandemic influenced the roll out of the NEWS intervention in this area?

Methods

Semi-structured interviews were conducted with stakeholders, including care home staff, NHS healthcare professionals and commissioners, in a CCG area in the North-East of England. This area is one of the 20% most deprived in England with high rates of morbidity and a lower-than-average life expectancy, with a resulting pressure on care home bed availability. Stakeholders were identified using purposive sampling, through existing links with local NHS staff. Participants were also asked to identify other colleagues involved in the NEWS intervention in this area (snowball sampling). We sought to include staff from a range of residential and nursing care homes, particularly with varied experience using NEWS, including long-standing users and users who received the intervention as part of the accelerated roll-out in response to COVID-19. A key NHS employee, known to potential participants, acted as a gatekeeper, sending introductory emails with a participant information sheet. Once consent to be contacted by the research team was secured, a researcher RS, made direct contact by email to invite participation. All individuals approached agreed to take part. Informed consent was secured either electronically or verbally in line with Health Research Authority principles.

All interviews were carried out remotely by telephone or videoconferencing, during May 2020. Development of an interview topic guide (Box 2) was informed by previous research conducted by the team, published literature evaluating the use of NEWS in care homes, and relevant literature and direct communications to the research team describing care home experiences of the pandemic. Field notes were taken after interviews.

Box 2. Interview topics

For peer review only - http://bmjopen.bmj.com/site/about/guidelines.xhtml

- Perceived validity of NEWS in care homes
- Experiences of using NEWS before, and during the pandemic
- NEWS training for care homes
- Perceptions on the usefulness of NEWS during the pandemic, including identification of potential cases
- How NEWS influenced decision making and triage, within and outwith care homes
- Variations in use of NEWS during the pandemic

Interviews were carried out by RS or SR (both female Research Associates, doctorates in health services research and medical sociology, and experience of qualitative studies in care homes), audio recorded and transcribed verbatim. All transcripts were anonymised. Interviews lasted 30-60 minutes. Approval was granted by Newcastle University Faculty of Medical Sciences Research Ethics Committee (ref: 2569/2020) and NHS North of England Commissioning Support; HRA approvals were not needed as the work was deemed to be a service evaluation.

Patient and public involvement

Patients and the public were first involved in this study at the idea generation stage, via the Newcastle University Care Home Interest PPI Group (CHIG). The research questions, topic guide, and study design were discussed via email with group members before the study commenced. CHIG members stressed the importance of carrying out interviews remotely and flexibly to time constraints, given the burden of the pandemic on the health and social care sector. Our discussions with this PPI group confirmed that COVID-19 related research in care homes is viewed as a pressing research priority. PPI partners will be consulted on appropriate dissemination strategies.

Data analysis

RS and SR conducted a thematic analysis of the dataset, following the principles of Braun and Clark's six-phase framework[18]. An inductive, data-driven approach was taken to analysis, meaning that the themes generated through our analysis emerged from the data itself, rather than via the use of a pre-existing coding frame.[19] We chose this approach to collect rich data on our topic, using a contextualist approach which "acknowledges the ways individuals make meaning of their experience, and, in turn, the ways the broader social context impinges on those meanings, while retaining focus on the material and other limits of 'reality'." [18] (p. 9) NVivo 11 software was used to aid in data management.

For peer review only - http://bmjopen.bmj.com/site/about/guidelines.xhtml

Transcripts were read in full (phase 1 – familiarisation with data), then coded line by line, separately, by RS and SR (phase 2 – generating initial codes). Codes were compared within and between transcripts, using the constant comparative technique[20], to streamline the coding framework and identify themes (phase 3 - search for themes). Emergent themes were discussed within the study team (phase 4 – reviewing themes), and linked together to form a final set of themes and sub-themes (phase 5 – defining themes) which were then situated within each of the three key research questions for the purposes of reporting (phase 6 – writing up analysis). Interviews ceased when no new themes were identified from the data (data saturation) as agreed by RS and SR.

Results

We interviewed 17 stakeholders, comprising 10 from the care home sector across 7 care homes, and 7 from the NHS. See Table 1. Of the 7 care homes, 5 provided both residential and nursing care, and 2 provided solely residential care. See Table 2 for care home characteristics. There are no care homes in this area which provide nursing care only. One care home received the accelerated form of the NEWS training and implementation during the COVID-19 pandemic, the remainder had had NEWS implemented pre-pandemic.

Table 1. Interviewees and their working role

Working role	Sector	n	Description
**Care home manager**<br>***Nursing background***<br>***Non-nursing background***	Care home	7<br>*(3)*<br>*(4)*	Managers oversee the running of the home ensuring that guidelines and regulations are adhered to.
**Care home senior carer/nurse**	Care home	3	Senior carers provide care and limited healthcare to residents and oversee the care provided by junior carers.<br>Care home nurses provide healthcare to residents.
**Director**	NHS	1	Director within a regional NHS Foundation Trust. Provided advice to the implementation team on the use of NEWS within the context of patient safety and reducing avoidable hospital admissions.

For peer review only - http://bmjopen.bmj.com/site/about/guidelines.xhtml

**Director**	NHS-related body	1	Supported the implementation team and aided networking and learning between various CCGs and academic institutions.
**NHS nurses**<br>***Specialist Older Persons' Nurse***<br>***Clinical Educator***	NHS	4<br>*(3)*<br>*(1)*	Specialist Nurses visit care homes to provide healthcare to care home residents. They provide a link between the care homes and external services, aiming to prevent avoidable hospital admissions. The Clinical Educator provided training to the care homes as well as ongoing support and monitoring.
**Commissioning of NHS services for older people**	NHS	1	Programme manager with a focus on technological innovation in care settings leading the implementation of NEWS in care homes.
	**Total:**	**17**	

Table 2. Care home characteristics.

Care home	Care provided	Number of beds
**Care home 1**	General care and specialist dementia	~50
**Care home 2**	General care and specialist dementia	~50
**Care home 3**	General care and specialist dementia	~30
**Care home 4**	Predominately specialist dementia	~40
**Care home 5**	Specialist dementia and end of life care	~40
**Care home 6**	Specialist dementia	~40
**Care home 7**	General care and nursing	~30

Higher level themes are organised according to how they address each of the key research questions, which are presented in turn.

1. **How have care home staff used the NEWS intervention to assess resident illness during the COVID-19 pandemic?**

For peer review only - http://bmjopen.bmj.com/site/about/guidelines.xhtml

The NEWS intervention during the pandemic: an adjunct to COVID-19 identification

Many stakeholders described how, prior to the COVID-19 outbreak, the use of the NEWS package had become embedded and habitualised into the everyday routines of care homes, and staff interactions with external healthcare services. Care homes recorded NEWS baseline scores regularly (for routine monitoring and comparison when residents became acutely unwell), except for those near the end of life. A NEWS was frequently requested by community healthcare professionals responding to concerns from care homes, and staff often calculated a NEWS before contacting healthcare professionals.

At the outbreak of the COVID-19 pandemic in the UK and the start of lockdown measures, use of the NEWS by care homes in this area increased substantially. Using NEWS became increasingly embedded into daily care home and NHS practices, and was viewed as a key aspect of the pandemic response in this setting. Care home staff, NHS healthcare professionals and service commissioners shared a belief that measuring NEWS, its component physiological observations, alongside the associated education and support package, have enhanced the response to deteriorating resident health during the pandemic.

Taking vital signs observations and using NEWS with the specific intention of identifying possible COVID-19 infection varied across the care homes in our study. Stakeholders highlighted the power of the NEWS in identifying early illness due to COVID-19 as well as other causes, and the ability of the NEWS intervention to facilitate remote monitoring, thereby decreasing the need for healthcare professionals to visit care homes.

The use of individual physiological observations to identify COVID-19

If COVID-19 was suspected, care home staff frequently measured temperature and oxygen saturations to use as individual physiological measures, as opposed to the NEWS. The use of these physiological observations for identifying COVID-19 disease was advocated by a local NHS network. Care home staff appreciated the importance of responding to isolated abnormalities in temperature or oxygen saturations to identify COVID-19, even if the NEWS was not triggering a high level of concern. Stakeholders remained broadly sceptical about the sensitivity and specificity of these clinical observations to accurately identify COVID-19 in this population – feeling that they went some way to help, and could prompt further investigation of potential COVID-19, but could not be conclusive.

For peer review only - http://bmjopen.bmj.com/site/about/guidelines.xhtml

[There are] two main symptoms with our experience, high temperature and low oxygen saturations. We've found in a couple of our residents, […] they had no temperature, and no breathing difficulty, but I couldn't understand - and when we checked their NEWS score we found oxygen saturation was low. So it helps us in the early diagnosis and to do appropriate support for the residents. Care home manager 1

The response to the infection is hugely physiological. So NEWS is a way of prompting you to do physiology. […] I think if you'd asked me six months ago I'd have said 'Actually the NEWS score is more useful than the underlying physiology.' Actually, I'd be tempted to go the other way around now and say 'You do a NEWS score because it makes you look at the physiology and you're going to go, oh my god, look at that oxygen saturation.' NHS director

Frequency of NEWS recordings and individual observations significantly increased month-on-month from March 2020 onwards, with some care homes taking baseline NEWSs several times a week. Other care homes recorded the NEWS sporadically, suggesting wide variation in the use of NEWS in this area during the pandemic. Figure 1 illustrates the increase in NEWS and individual observations recording before and during the pandemic.

FIGURE 1 HERE

Using the NEWS intervention to remotely monitor health

It was felt among care home and NHS healthcare professionals that, prior to the pandemic, NEWS could speed up initial triage and facilitate remote treatment decisions, expediting the process for residents to receive the care they required. Referrals accompanied by a NEWS score reduced the need for clinicians to visit care homes to take physical observations. Identification of early illness was considered to be more efficient with the use of NEWS. Set within this context, it is perhaps not surprising that another positive aspect of the NEWS intervention during the pandemic was to facilitate remote monitoring of residents' health by healthcare professionals. This was believed to have reduced unnecessary footfall and thus infection risk, which was a major driver to the accelerated roll out of the NEWS intervention during the pandemic.

For peer review only - http://bmjopen.bmj.com/site/about/guidelines.xhtml

> *We had to just take a stand and reflect and say, 'What do we need to do? We know that the predictions [are that COVID-19] is going to come. We're likely to have to go into lockdown. Nobody is going to be able to go into care homes.' So we thought, 'actually NEWS is an important tool in that. […] We just need to respond'* NHS commissioner

> *CCGs have been formally told to set up remote MDT care home rounds, so they are having to put the IT in place. […] You need a digital way of recording [resident] observations, and then you can put in a safety netting process that will allow the care home to get back in touch. Because there's going to be loads of [residents] where you're going to go, 'they're not quite right but I definitely don't want to send them to hospital.'* NHS director

The ability for NHS staff, such as community nurses, to remotely access physiological observations and compare with baselines, was of particular value. The camera facility of the NEWS tablet was also unlocked in response to the pandemic, giving external healthcare professionals the ability to assess and treat wounds remotely.

> *[int: Are there any doctors or GPs who are taking it upon themselves to log on to the [cloud system] and look at the NEWS scores?] Yes, absolutely. [Pre-pandemic] I don't think it was utilised [by them], especially not as much as it could have been, but due to the COVID crisis, everyone across the city now want access to it and it's obviously taken a pandemic to realise how valuable the tool is.* NHS specialist nurse 1

> *I think the biggest change for the [use of the NEWS intervention] at the moment is the pictures. […] For example, if there was a patient who had a skin flap […] We'll say, 'Will you take a photo? Will you upload it so we can see it?' We can physically see that photo and then we can assess the severity, we can assess when the visit should next take place […] it's brilliant for that aspect.* NHS specialist nurse 2

2. What is the experience of care home staff of using the NEWS intervention during the COVID-19 pandemic?

Empowerment of care home staff with the NHS agenda and language

For peer review only - http://bmjopen.bmj.com/site/about/guidelines.xhtml

Using the NEWS intervention to help identify and triage responses to deteriorating resident health was described by stakeholders as empowering. For care homes, the NEWS intervention represented a helpful link to the NHS. The NEWS intervention represented 'hard clinical data' which care home staff could generally rely upon to back up their intuition that the resident was unwell (termed 'soft signs' e.g. decreased appetite). Having a common clinical language which care home staff could use – or leverage for action, where necessary – was useful to promote integrated working between care homes and the NHS. It also facilitated triage to appropriate services, often negating the need for a primary care healthcare professional to attend in person, which is an important consideration during the pandemic.

Care home staff felt confident that with a NEWS reading, their concerns about deteriorating residents would be taken seriously by external healthcare professionals, as it evidenced their concerns. This was compared positively to care home staff's previous experiences seeking help from primary care, where staff often felt that concerns were not listened to. This feeling of empowerment was capitalised upon by both care homes and local NHS services during the COVID-19 pandemic.

> *When we've rang the likes of home recovery or 111 and we're challenged as to why we're ringing, when we can't really put a finger on it that somebody's not well. Now, this [NEWS] has really aided us to be able to look for those signs, look for those symptoms, using the NEWS score and being able to give better communication across the telephone as to why and what has happened with that particular resident. I know there's been times where we've kind of been brushed off by services. And I'm a big believer in our care staff, they may not be qualified nurses but they know their residents sometimes better than we do. […] Now I feel like we've got this tool now to be able to kind of prove that somewhat as well, and it does help back up what we're saying when it comes to our residents. So it's been great.* Care home manager 2

During the COVID-19 pandemic, care home staff were relied to take on clinical procedures and observations usually carried out by visiting NHS professionals. This challenge to traditional role boundaries was welcomed and encouraged by NHS stakeholders, as it met the needs of the NHS to reduce footfall into care homes and limit the number of hospital admissions. In the early days of the pandemic, and without straightforward guidance for care homes, care home staff also welcomed this extension to their roles. They were able to measure and focus on clinical observations – in effect, the NEWS intervention facilitated a more concrete response within care homes to tackling a then-unknown virus.

For peer review only - http://bmjopen.bmj.com/site/about/guidelines.xhtml

[int: What do you think about the use of NEWS during this pandemic in care homes?] Well I've always loved it. I really love the NEWS tablet and for why, because I think it totally empowers the care staff. […] Care staff, when they say, 'They're just not right. I don't know what's wrong with them, but the person's not right.' Now they've got a little more ammunition to say, 'well actually, such-and-such isn't very well. These are the observations…' and it makes them have more of a voice. That's what I love about it so when they ring the GP and they say, 'Well actually the observations are blah, blah, blah,' and they're almost listened to a little bit more because they've got some sort of clinical information rather than just saying, 'Oh I don't know what's wrong with them.' I think that applies to here and now as well with the pandemic. NHS specialist nurse 2

3. How has the COVID-19 pandemic influenced the roll out of the NEWS intervention?

Centrality of training relationships and clinical support for accelerated implementation of NEWS during a pandemic

When the pandemic broke out, the CCG commenced a rapid roll out of the NEWS intervention to the minority of care homes in the area that were waiting for implementation. Care homes already using the NEWS intervention before the pandemic were contacted by the clinical educator to reinforce that support was available. The pandemic highlighted the importance of care homes, particularly those without registered nursing staff, having an engaged and supportive nominated NHS clinical educator.

Training and implementing the NEWS intervention in care homes during the pandemic

Training to support rapid implementation during the pandemic consisted of a short, socially distanced visit from the clinical educator to the care home, training one or two key members of staff in the home rather than a larger body of staff, and without a practical component. This rapid roll out model demonstrated that relationships built during training were integral to ongoing NEWS use and understanding. The training constraints related to the pandemic were perceived as having had a major impact on the quality of the rapid implementation and confidence of staff.

For peer review only - http://bmjopen.bmj.com/site/about/guidelines.xhtml

[During the rapid training], I don't feel like I built great rapport. I don't feel like it instilled that much confidence in them and I haven't really received many phone calls from those homes. Yes they're doing NEWS, but usually the homes that I've really been visibly in, really supported them and built those rapports and stuff up with, I feel like I get more phone calls which is great because they want support which then that in turn means they've picked up something, they're looking for answers and help and they get it. Where those other homes, I would worry that maybe they don't feel as confident to pick up the phone and speak with me because I've raced in, did what I had to do and then left – and it's very difficult to build up rapport over time, and trust. NHS specialist nurse 1

Ongoing clinical support for care homes during the pandemic

Emphasis was placed on the importance of ongoing clinical support for care homes. In this area, care homes had access to the clinical educator and a specialist nurse with a specific care home remit. Having these key contacts, who were knowledgeable and approachable, was valued by care homes and capitalised upon during the COVID-19 pandemic. They were able to build upon long-term relationships with each other, cultivating understanding of each sector and respect for each other's role and remit. This cross-sector team working helped to legitimise use of NEWS in a non-NHS setting, and smoothed many processes of integrated care over traditional NHS and social care boundaries.

This is not just about the technical or the medical aspect, it's often about the relationships that [clinical educator] is then building up. […] I think that methodology has worked because you're consolidating it with a really good grip and really getting [care homes] to be the expert. NHS commissioner

[Clinical educator] is a very approachable person. You can email him, you phone him and in all fairness, we would probably see [clinical educator] at least probably once a week. He'll pop in just to make sure everything is alright and we're well, you know, and if we're having any problems. So that support is continually there. Care home manager 4

Discussion

For peer review only - http://bmjopen.bmj.com/site/about/guidelines.xhtml

Summary of findings

The roll out of the NEWS intervention in the study area was accelerated by COVID-19. Care home staff and NHS commissioners viewed it as an important part of a combined care home-NHS response to care delivery during the pandemic. The NEWS intervention was used as an adjunct to carer identification of deteriorating resident health, whether due to COVID-19 or other acute illnesses. It was perceived to have facilitated remote decision-making by healthcare and care home staff and minimised footfall in care homes during the pandemic. In relation to possible COVID-19 infection, care home staff reported increased reliance on specific physiological measures such as oxygen saturations, as opposed to the overall NEWS. Care home staff felt empowered by their extended role in the measurement of physiological observations; better able to respond to resident deterioration and communicate their concerns to healthcare professionals. However, this study did not provide insights into which were the active ingredients in the NEWS intervention. The intervention encompassed training and support from a clinical educator and sharing of clinical measurements and NEWS with health services. Dis-entangling the relative contributions of education, technology and clinical assessment to resident care is challenging.

Comparison with other work

Previous evaluations of the use of NEWS in a non-hospital setting reflect many of our findings, including the benefits of using a common clinical language, and facilitating communication between sectors.[21] The importance of using NEWS as a part of a wider assessment of health, rather than a ritualistic, task-oriented procedure[22], has been stressed by both the UK Royal College of Physicians[9] and those evaluating its use in other settings including primary[13] and community care.[21] The clinician-led training and ongoing support model for the NEWS intervention in place in this area addressed these concerns.

There is some evidence to support the use of NEWS in the identification of and triage of hospital patients with suspected COVID-19.[23 24] However, there have been calls for NEWS to be modified with a more sensitive score for oxygen demand to better account for the development of hypoxic respiratory failure in COVID-19 patients.[25] Evidence for the use of NEWS in community settings during the pandemic remains sparse. There is little comparable evidence available to evaluate the utility of NEWS in care homes specifically during the COVID-19 pandemic.

This intervention led to an increase in assessment and monitoring of a vulnerable population of care home residents during a global pandemic. Greater vigilance and attention to physiological measures

For peer review only - http://bmjopen.bmj.com/site/about/guidelines.xhtml

within care homes may be the critical component, irrespective of the detail of NEWS and adherence to an escalation plan. However, this intervention has a number of features that minimised face to face contacts with residents, which should reduce infection risk for residents and staff. This is important, as a high proportion of care home residents are asymptomatic when they test positive for COVID-19[26], a finding reflected in the experiences of our interviewees. Bluetooth transfer of data from measuring equipment to digital tablet, cloud data storage and the possibility of rapid information transfer to outside agencies, were all of potential benefit during COVID-19. The lack of any requirement to manually input recordings from thermometers and blood pressure monitors was a time saving feature, that promoted accuracy and uptake[27], and in the pandemic, minimised time in close contact with the resident. It is also noteworthy that during COVID-19, staff often completed a subset of measurements, rather than a complete assessment. Selection of what were perceived to be the most important measures (oxygen saturation and temperature) was a modification of NEWS that further reduced staff resident contact. It is also supported by recent evidence[28] that highlights the potential utility of individual oxygen saturation, respiratory rate, and temperature measurements. However, reducing the range of measurements does mean that a NEWS cannot be calculated. Whether the absence of NEWS has any impact on resident care, is an important question for the future.

From an implementation perspective, the training and support for the NEWS intervention was delivered by an experienced NHS nurse who understood the needs of the sector. In terms of work needed to implement change[27], the organisational burden of planning and implementation for the CCG and NHS clinical educator was minimised as they had already been actively engaged in a revised implementation prior to the pandemic and thus there was a pre-existing awareness and embeddedness of NEWS across the care home sector in the region. Most care homes in our sample were already familiar with NEWS prior to the pandemic, hence, a shift in roles and identities had already taken place and only minimal work was needed to engage care home staff in implementation[27] or to change working practices, after the outbreak. This pre-existing familiarity, coupled with the reported ease of use of the NEWS equipment and the support from the wider healthcare system, enhanced the adoption, spread, and scale-up[27] of the NEWS intervention in these care homes during the pandemic.

Regular, informal contact from a trusted NHS source to care homes may have been particularly important early in the pandemic, when information was sparse and there were many uncertainties about how best to provide care.[29] The focus in training on identifying 'soft signs' of deterioration was also significant. So called 'soft signs' – a resident having a reduced appetite for a favourite food, for example - are not perceived as measurable or objective. However, they are observed by care home

For peer review only - http://bmjopen.bmj.com/site/about/guidelines.xhtml

staff, and have their own unique importance.[30] Inclusion within the training could be seen as a validation of care home staff contribution to resident care.

Implementation of change in the care home sector is challenging. Resources are limited and pressures on staff in particular, may limit the potential to engage in new initiatives.[31]Engaging and supporting care homes, with a good understanding of their needs, is critical when implementing change.[16 32] In this case, the intervention was introduced from outside the sector, raising the possibility that it would be perceived as irrelevant or inappropriate. Pre-pandemic and ongoing NHS support for the NEWS intervention legitimised and enhanced its use, reflecting other studies in the care home sector.[33] Widespread changes to working practices during COVID-19 are also likely to have had a major influence on staff readiness to embrace change.

Strengths and limitations

We were able to interview ten care home staff from seven care homes in the locality, and seven NHS staff, despite the pandemic related pressure on the NHS and social care sectors during the interviewing period (May 2020). This represents a strength of our study and highlights the importance that interviewees placed on the topic and expressing their views. However, our data may not be representative of all care home experiences in other areas of the country, particularly those who are not working with NEWS. Similar interventions have been introduced across England, but this model is specific to the study area, which makes comparison with cross-sector interventions in other geographical areas more difficult.

We were unable to recruit GPs, which we believe was due to the pressure the pandemic had placed on primary care. The pandemic visiting restrictions in care homes also meant that we were unable to interview any care home residents themselves to explore their experiences during the pandemic and their understanding of care had been influenced by the introduction of the NEWS intervention. Future work should aim to include these groups, so that their views are directly included rather than inferred.

Conclusion

The NEWS intervention may have a useful role in care homes during the COVID-19 pandemic, enhancing remote working and offering staff some reassurance and structure to their role. However, it is important to acknowledge the paucity of data on NEWS in care homes, despite increasing uptake. Positive staff perceptions from this study now need to be supplemented with data on the impact on

For peer review only - http://bmjopen.bmj.com/site/about/guidelines.xhtml

resident health and wellbeing, workload, and service utilisation, during the COVID-19 pandemic and beyond. A multidisciplinary consensus on best practice for NEWS use in this sector is required.

Contributors

RS, SR and JA designed the study with JL and BH. RS and SR conducted interviews. RS, SR, and JA analysed data. RS, SR, JA and BH drafted the article, and AR, JL, JG, and RB performed critical revision of the article for important intellectual content. RS is guarantor of the paper. All authors approved the final version to be published.

Data sharing statement

Data are not publicly available.

Word count

5381 words.

Declaration of Conflicts of Interest

The authors declare no conflicts of interest.

Declaration of Funding

This study is funded by the National Institute for Health Research (NIHR), Research for Patient Benefit [award number PB-PG-0418-20034]. JL is funded by the NIHR Applied Research Collaboration North East and North Cumbria (ARC NENC). The views expressed are those of the author(s) and not necessarily those of the NIHR or the Department of Health and Social Care.

References

1. Competition and Markets Authority. Care home market study summary of final report. London, 2017.
2. Jarrett T. Social care: care home market – structure, issues, and cross-subsidisation. London: House of Commons Library, 2018.
3. Gordon AL, Franklin M, Bradshaw L, et al. Health status of UK care home residents: a cohort study. Age and Ageing 2014;**43**(1):97-103.
4. Office for National Statistics. Deaths registered weekly in England and Wales, provisional 2020 [Available from: https://www.ons.gov.uk/peoplepopulationandcommunity/birthsdeathsandmarriages/deaths/datasets/weeklyprovisionalfiguresondeathsregisteredinenglandandwales.
5. Office for National Statistics. Coronavirus (COVID-19) related deaths by occupation, England and Wales: deaths registered up to and including 20 April 2020 2020 [Available from:

For peer review only - http://bmjopen.bmj.com/site/about/guidelines.xhtml

https://www.ons.gov.uk/releases/covid19relateddeathsbyoccupationenglandandwalesdeathsregistereduptoandincluding20thapril2020.
6. World Health Organization. Statement – Invest in the overlooked and unsung: build sustainable people-centred long-term care in the wake of COVID-19 2020 [Available from: http://www.euro.who.int/en/media-centre/sections/statements/2020/statement-invest-in-the-overlooked-and-unsung-build-sustainable-people-centred-long-term-care-in-the-wake-of-covid-19.
7. Brainard J, Rushton S, Winters T, et al. Introduction to and spread of COVID-19-like illness in care homes in Norfolk, UK. Journal of Public Health 2020.
8. NHS England. NHS Five Year Forward View. London, 2014.
9. Royal College of Physicians. National Early Warning Score (NEWS) 2: Standardising the assessment of acute-illness severity in the NHS. Updated report of a working party. London: RCP, 2017.
10. West Hampshire Clinical Commissioning Group. RESTORE2: recognise early soft-signs, take observations, respond, escalate 2019 [Available from: https://www.westhampshireccg.nhs.uk/restore2-training-and-resources.
11. Hodgson P, Cook G, Thompson J, et al. Assessment and clinical decision making of the acutely ill older care home resident: implementation of NEWS in Gateshead Care Homes. Report., 2017.
12. Finnikin S, Hayward G, Wilson F, et al. Are referrals to hospital from out-of-hours primary care associated with National Early Warning Scores? Emergency Medicine Journal 2020;**37**(5):279-85.
13. Greenhalgh T, Treadwell J, Burrow R, et al. Should we use the NEWS (or NEWS2) score when assessing patients with possible COVID-19 in primary care?: Oxford COVID-19 Evidence Service, 2020.
14. Barker RO, Stocker R, Russell S, et al. Distribution of the National Early Warning Score (NEWS) in care home residents. Age and Ageing 2019;**49**(1):141-45.
15. Hodge S, Thompson C, Gordon AL. National early warning scores in care homes: do policy imperatives reflect a genuine need? Age and Ageing 2019;**49**(1):5-6.
16. Russell S, Stocker R, Barker RO, et al. Implementation of the National Early Warning Score in UK care homes: a qualitative evaluation. British Journal of General Practice 2020;**70**(700):e793-e800.
17. British Geriatrics Society. COVID-19: Managing the COVID-19 pandemic in care homes for older people. Good Practice Guide 2020 [Available from: https://www.bgs.org.uk/resources/covid-19-managing-the-covid-19-pandemic-in-care-homes#anchor-nav-identifying-residents-who-may-have-covid-19-and-how-to-respond.
18. Braun V, Clarke V. Using thematic analysis in psychology. Qualitative research in psychology 2006;**3**(2):77-101.
19. Braun V, Clarke V. Reflecting on reflexive thematic analysis. Qualitative Research in Sport, Exercise and Health 2019;**11**(4):589-97.
20. Glaser BG. The constant comparative method of qualitative analysis. Social problems 1965;**12**(4):436-45.
21. Brangan E, Banks J, Brant H, et al. Using the National Early Warning Score (NEWS) outside acute hospital settings: a qualitative study of staff experiences in the West of England. BMJ Open 2018;**8**(10):e022528.
22. Massey D, Chaboyer W, Anderson V. What factors influence ward nurses' recognition of and response to patient deterioration? An integrative review of the literature. Nursing Open 2017;**4**(1):6-23.
23. Myrstad M, Ihle-Hansen H, Tveita AA, et al. National Early Warning Score 2 (NEWS2) on admission predicts severe disease and in-hospital mortality from Covid-19 – a prospective cohort study. Scandinavian Journal of Trauma, Resuscitation and Emergency Medicine 2020;**28**(1):66.

For peer review only - http://bmjopen.bmj.com/site/about/guidelines.xhtml

24. Gidari A, De Socio GV, Sabbatini S, et al. Predictive value of National Early Warning Score 2 (NEWS2) for intensive care unit admission in patients with SARS-CoV-2 infection. Infectious Diseases 2020;**52**(10):698-704.
25. Lim NT, Pan D, Barker J. NEWS2 system requires modification to identify deteriorating patients with COVID-19. Clinical Medicine 2020;**20**(4):e133-e34.
26. Graham NSN, Junghans C, Downes R, et al. SARS-CoV-2 infection, clinical features and outcome of COVID-19 in United Kingdom nursing homes. Journal of Infection 2020.
27. Greenhalgh T, Wherton J, Papoutsi C, et al. Beyond Adoption: A New Framework for Theorizing and Evaluating Nonadoption, Abandonment, and Challenges to the Scale-Up, Spread, and Sustainability of Health and Care Technologies. J Med Internet Res 2017;**19**(11):e367-e67.
28. Stow D BR, Matthews FE, Hanratty B. . National Early Warning Scores and COVID-19 deaths in care homes: a longitudinal ecological study. BMJ Open 2020;**In press**.
29. Spilsbury K, Devi R, Griffiths A, et al. SEeking AnsweRs for Care Homes during the COVID-19 pandemic (COVID SEARCH). Age and Ageing 2020.
30. Tingström P, Milberg A, Sund-Levander M. Early nonspecific signs and symptoms of infection in institutionalized elderly persons: perceptions of nursing assistants. Scand J Caring Sci 2010;**24**(1):24-31.
31. Goodman C, Dening T, Gordon AL, et al. Effective health care for older people living and dying in care homes: a realist review. BMC Health Services Research 2016;**16**:269.
32. Stocker R, Bamford C, Brittain K, et al. Care home services at the vanguard: a qualitative study exploring stakeholder views on the development and evaluation of novel, integrated approaches to enhancing healthcare in care homes. BMJ Open 2018;**8**(3):e017419.
33. Gordon AL, Goodman C, Davies SL, et al. Optimal healthcare delivery to care homes in the UK: a realist evaluation of what supports effective working to improve healthcare outcomes. Age and Ageing 2018;**47**(4):595-603.

For peer review only

For peer review only - http://bmjopen.bmj.com/site/about/guidelines.xhtml

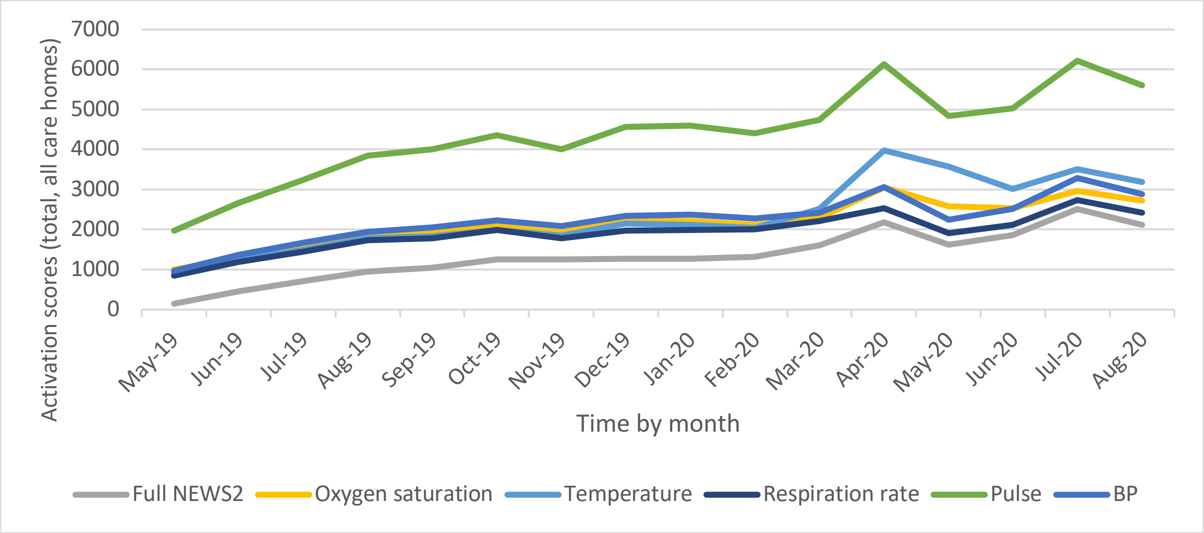

Figure 1 Frequency of NEWS2 and its component measures taken in care homes in the study area

For peer review only

Manuscript: Remote assessment of care home residents during the COVID-19 pandemic – what is the role of the National Early Warning Score? A qualitative interview study

Consolidated criteria for reporting qualitative studies (COREQ): 32-item checklist

Developed from:
Tong A, Sainsbury P, Craig J. Consolidated criteria for reporting qualitative research (COREQ): a 32-item checklist for interviews and focus groups. *International Journal for Quality in Health Care*. 2007. Volume 19, Number 6: pp. 349 – 357

No. Item	Guide questions/description	Reported on Page #
**Domain 1: Research team and reflexivity**		
Personal Characteristics		
1. Inter viewer/facilitator	Which author/s conducted the inter view or focus group?	RS and SR, p5
2. Credentials	What were the researcher's credentials? E.g. PhD, MD	PhDs, p5
3. Occupation	What was their occupation at the time of the study?	Research Associates, p5
4. Gender	Was the researcher male or female?	Both female, p5
5. Experience and training	What experience or training did the researcher have?	PhDs and significant experience in care home related qualitative research. P5
Relationship with participants		
6. Relationship established	Was a relationship established prior to study commencement?	No
7. Participant knowledge of the interviewer	What did the participants know about the researcher? e.g. personal goals, reasons for doing the research	Nothing other than the study information
8. Interviewer characteristics	What characteristics were reported about the inter viewer/facilitator? e.g. Bias, assumptions, reasons and interests in the research topic	None relevant

For peer review only

**Domain 2: study design**		
Theoretical framework		
9. Methodological orientation and Theory	What methodological orientation was stated to underpin the study? e.g. grounded theory, discourse analysis, ethnography, phenomenology, content analysis	Phenomenology, p4
Participant selection		
10. Sampling	How were participants selected? e.g. purposive, convenience, consecutive, snowball	Purposive and snowball sampling, p4
11. Method of approach	How were participants approached? e.g. face-to-face, telephone, mail, email	Email, p4
12. Sample size	How many participants were in the study?	17, p5
13. Non-participation	How many people refused to participate or dropped out? Reasons?	No refusals or drop outs, p4
Setting		
14. Setting of data collection	Where was the data collected? e.g. home, clinic, workplace	Home and workplace, p4-5
15. Presence of non-participants	Was anyone else present besides the participants and researchers?	No, p4
16. Description of sample	What are the important characteristics of the sample? e.g. demographic data, date	Information and table on p4-5
Data collection		
17. Interview guide	Were questions, prompts, guides provided by the authors? Was it pilot tested?	Yes, p5
18. Repeat interviews	Were repeat inter views carried out? If yes, how many?	No
19. Audio/visual recording	Did the research use audio or visual recording to collect the data?	Audio recording, p5
20. Field notes	Were field notes made during and/or after the inter view or focus group?	Afterwards, p5
21. Duration	What was the duration of the inter views or focus group?	30-60 minutes, p5
22. Data saturation	Was data saturation discussed?	Yes, p6
23. Transcripts returned	Were transcripts returned to participants for comment and/or correction?	No
**Domain 3: analysis and findings**		
Data analysis		
24. Number of data coders	How many data coders coded the data?	2, p6

For peer review only

25. Description of the coding tree	Did authors provide a description of the coding tree?	No (only themes and sub-themes, p6 onwards)
26. Derivation of themes	Were themes identified in advance or derived from the data?	Derived from the data, p6
27. Software	What software, if applicable, was used to manage the data?	NVivo, p5
28. Participant checking	Did participants provide feedback on the findings?	No
Reporting		
29. Quotations presented	Were participant quotations presented to illustrate the themes/findings? Was each quotation identified? e.g. participant number	Yes, p6 onwards
30. Data and findings consistent	Was there consistency between the data presented and the findings?	Yes
31. Clarity of major themes	Were major themes clearly presented in the findings?	Yes
32. Clarity of minor themes	Is there a description of diverse cases or discussion of minor themes?	Yes

For peer review only

BMJ Open

Experiences of a National Early Warning Score (NEWS) intervention in care homes during the COVID-19 pandemic: A qualitative interview study

Journal:	*BMJ Open*
Manuscript ID	bmjopen-2020-045469.R2
Article Type:	Original research
Date Submitted by the Author:	03-Jul-2021

Complete List of Authors:	Stocker, Rachel; Newcastle University, Population Health Sciences Institute<br>Russell, Sian; Newcastle University, Population Health Sciences Institute<br>Liddle, Jennifer; Newcastle University, Population Health Sciences Institute<br>Barker, Robert; Newcastle University, Population and Health Sciences Institute<br>Remmer, Adam; South Tyneside and Sunderland NHS Foundation Trust<br>Gray, Joanne; Northumbria University,<br>Hanratty, Barbara; Newcastle University, Population Health Sciences Institute<br>Adamson, Joy; University of York, Department of Health Sciences

**Primary Subject Heading**:	Geriatric medicine
Secondary Subject Heading:	Communication, General practice / Family practice, Health services research, Public health, Qualitative research
Keywords:	COVID-19, GERIATRIC MEDICINE, Organisation of health services < HEALTH SERVICES ADMINISTRATION & MANAGEMENT, Quality in health care < HEALTH SERVICES ADMINISTRATION & MANAGEMENT, PRIMARY CARE, QUALITATIVE RESEARCH

SCHOLARONE™
Manuscripts

I, the Submitting Author has the right to grant and does grant on behalf of all authors of the Work (as defined in the below author licence), an exclusive licence and/or a non-exclusive licence for contributions from authors who are: i) UK Crown employees; ii) where BMJ has agreed a CC-BY licence shall apply, and/or iii) in accordance with the terms applicable for US Federal Government officers or employees acting as part of their official duties; on a worldwide, perpetual, irrevocable, royalty-free basis to BMJ Publishing Group Ltd ("BMJ") its licensees and where the relevant Journal is co-owned by BMJ to the co-owners of the Journal, to publish the Work in this journal and any other BMJ products and to exploit all rights, as set out in our licence.

The Submitting Author accepts and understands that any supply made under these terms is made by BMJ to the Submitting Author unless you are acting as an employee on behalf of your employer or a postgraduate student of an affiliated institution which is paying any applicable article publishing charge ("APC") for Open Access articles. Where the Submitting Author wishes to make the Work available on an Open Access basis (and intends to pay the relevant APC), the terms of reuse of such Open Access shall be governed by a Creative Commons licence – details of these licences and which Creative Commons licence will apply to this Work are set out in our licence referred to above.

Other than as permitted in any relevant BMJ Author's Self Archiving Policies, I confirm this Work has not been accepted for publication elsewhere, is not being considered for publication elsewhere and does not duplicate material already published. I confirm all authors consent to publication of this Work and authorise the granting of this licence.

For peer review only

Experiences of a National Early Warning Score (NEWS) intervention in care homes during the COVID-19 pandemic: A qualitative interview study

Rachel Stocker[1,3], Siân Russell[1], Jennifer Liddle[1], Robert Oliver Barker[1], Adam Remmer[2], Joanne Gray, Barbara Hanratty[1], Joy Adamson[3]

1. Population Health Sciences Institute, Newcastle University
2. South Tyneside and Sunderland NHS Foundation Trust
3. Department of Health Sciences, University of York

Address correspondence to Dr Rachel Stocker, Population Health Sciences Institute, Newcastle University, Level 2, Newcastle Biomedical Research Building Campus for Ageing and Vitality, Newcastle upon Tyne NE4 5PL, UK.

Email: rachel.stocker@newcastle.ac.uk

For peer review only

Abstract

Background

The COVID-19 pandemic has taken a heavy toll on the care home sector, with residents accounting for up to half of all deaths in Europe. The response to acute illness in care homes plays a particularly important role in the care of residents during a pandemic. Digital recording of a National Early Warning Score (NEWS), which involves the measurement of physical observations, started in care homes in one area of England in 2016. Implementation of a NEWS intervention (including equipment, training and support) was accelerated early in the pandemic, despite limited evidence for its use in the care home setting.

For peer review only - http://bmjopen.bmj.com/site/about/guidelines.xhtml

Objectives

To understand how a National Early Warning Score (NEWS) intervention has been used in care homes in one area of North-East England during the COVID-19 pandemic, and how it has influenced resident care, from the perspective of stakeholders involved in care delivery and commissioning.

Methods

A qualitative interview study with care home (n=10) and NHS (n=7) staff. Data were analysed using thematic analysis.

Results

Use of the NEWS intervention in care homes in this area accelerated during the COVID-19 pandemic. Stakeholders felt that NEWS, and its associated education and support package, improved the response of care homes and healthcare professionals to deterioration in residents' health during the pandemic. Healthcare professionals valued the ability to remotely monitor resident observations, which facilitated triage and treatment decisions. Care home staff felt empowered by NEWS, providing a common clinical language to communicate concerns with external services, acting as an adjunct to staff intuition of resident deterioration.

Conclusions

The NEWS intervention formed an important part of the care home response to COVID-19 in the study area. Positive staff perceptions now need to be supplemented with data on the impact on resident health and wellbeing, workload, and service utilisation, during the pandemic and beyond.

Strengths and limitations of this study

- This study is the first to examine the role of a National Early Warning Score (NEWS) intervention in care homes during the COVID-19 pandemic in the UK.
- Care home and NHS staff were recruited and interviewed in May 2020, providing an important insight into the COVID-19 response in this sector.
- The role of NEWS in identifying COVID-19 disease and monitoring resident health, was explored in-depth with semi-structured remotely conducted interviews.
- General practitioners and care home residents were not recruited for practical reasons; future research should directly seek rather than infer their views.

For peer review only - http://bmjopen.bmj.com/site/about/guidelines.xhtml

For peer review only

Introduction

The care home sector is one of the most overlooked components of health and social care in the UK. Care homes provide accommodation, personal care and support, and companionship to some of the most vulnerable, frail, and medically complex older adults in society. There are more than 11,000 care homes (nursing and non-nursing) in the UK[1], home to over 400,000 residents, representing 16% of those aged 85 or over. The sector is dominated by private, for-profit, providers, who provide around four-fifths of care home beds in the UK.[2] Care homes may receive some funding from the NHS, however the majority of funding comes from local authorities, or from residents or residents' families. Care home residents receive medical care from visiting National Health Service (NHS) primary care professionals, including general practitioners and community nurses. Care configuration of NHS services for care home residents varies significantly across the country. Some areas provide a varied multidisciplinary healthcare team to support care home residents; others focus on specialist nurse provision; others have no special service.[3]

Care homes have been severely affected by the COVID-19 pandemic. The virus spread rapidly and many residents died in a short space of time. Estimates suggest that up to half of all COVID-19 related deaths in Europe were care home residents[4], and people working in social care are dying at twice the rate of the general population.[5] This situation has been described by the World Health Organisation as an "unimaginable human tragedy[6]". Many care homes have struggled with staffing, accessing personal protective equipment (PPE)[7], and maintaining isolation measures during the pandemic.

In recent years, efforts have been made to support the care home sector to improve the quality of health-related care.[8] Identifying and monitoring residents for early signs of acute illness, using tools such as the National Early Warning Score (NEWS; the latest version of the scoring system is the 'NEWS2'), has been a particular area of interest.[9] The NEWS was initially developed for use in hospital settings. It requires the measurement of six parameters: temperature, pulse, systolic blood pressure, respiratory rate, oxygen saturation and level of consciousness. The overall NEWS triggers a response, ranging from repeating the measurements within a specific time frame, to initiating an emergency medical response. The NEWS is intended to serve as an adjunct to, as opposed to replacing, a clinical decision.[9]

Implementation of the NEWS into care homes is already underway across multiple regions in the UK[10 11], despite a lack of validation and proven effectiveness outside the hospital setting.[12] The aim is to improve the response to acute illness in care home residents by improving triage and communication with external healthcare professionals, and to reduce avoidable hospital admissions. In care homes, the NEWS intervention has generally been implemented as a 'package', which includes as a minimum

For peer review only - http://bmjopen.bmj.com/site/about/guidelines.xhtml

the training and equipment to measure the vital observations needed to generate the NEWS score itself. Some implementations of a NEWS package have additionally included an ongoing support programme to care homes, and the use of a cloud-based storage system to store (and sometimes transmit to primary care staff for use in triage) NEWS scores. Concerns have been expressed about the appropriateness of using the NEWS in care homes, including potential adverse influences on palliative care.[13] NEWS was also designed for use by clinically trained staff, such as registered nurses, who are not on-site in residential care facilities. Previous work indicates that whilst the collection of NEWS in care homes appears to be feasible[14], the complexity of the care home context and subsequent intricacies in implementing NEWS need careful thought and planning to exploit any potential benefits for residents.[15 16] Early in the COVID-19 pandemic in the UK, the British Geriatrics Society emphasised the importance of care home staff having the skills, training, and equipment to identify deterioration in residents, and advocated the use of systems that incorporate NEWS.[17]

This study focuses on the ways in which one Clinical Commissioning Group (CCG) with a high level of socioeconomic deprivation in the North-East of England, utilised their NEWS intervention in response to the COVID-19 pandemic. Having initially implemented a NEWS intervention in 2016, providing care homes with the equipment for taking vital signs, a digital tablet for recording NEWS and associated training, in 2019 the CCG invested further when a nurse was appointed as a clinical educator to provide more tailored training and support to care home staff (see Box 1 for an outline of the NEWS intervention in this area). Whilst most care homes in the CCG had received the 2019 intervention, for those who had not the CCG accelerated the roll out of this, with the aim of ensuring all residential and nursing homes had the equipment and training necessary to measure residents' physical observations and calculate a NEWS.

Box 1: NEWS intervention

Pre-COVID-19

- Equipment for taking vital signs and digital tablet for measuring and recording NEWS.
- Education and support delivered by an NHS clinical educator with a nursing background covering: practical use of equipment, interpretation of individual vital signs and resulting NEWS scores, contextualising NEWS observations and underpinning scoring system with the importance of 'soft signs' of deterioration.
- Internet web-based cloud storage system, whereby NEWS scores are automatically uploaded from the tablet to the storage system. The NHS clinical educator and other NHS staff involved in care for each care home can access the web-based system to view NEWS scores.

Additional function in response to COVID-19

- Additional functionality offered by the equipment was unlocked thereby expanding the NEWS package in this area further - notably a picture-taking facility to upload pictures of wounds.

For peer review only - http://bmjopen.bmj.com/site/about/guidelines.xhtml

This paper presents a qualitative interview study with key stakeholders, with the aim of understanding how a National Early Warning Score (NEWS) intervention has been used in care homes during the COVID-19 pandemic in the. UK, and how it has influenced resident care. Specific research questions were:

1. How have care home staff used the NEWS intervention to assess resident illness during the COVID-19 pandemic?
2. What is the experience of care home staff of using the NEWS intervention during the COVID-19 pandemic?
3. How has the COVID-19 pandemic influenced the roll out of the NEWS intervention in this area?

Methods

Semi-structured interviews were conducted with stakeholders, including care home staff, NHS healthcare professionals and commissioners, in a CCG area in the North-East of England. This area is one of the 20% most deprived in England with high rates of morbidity and a lower-than-average life expectancy, with a resulting pressure on care home bed availability. Stakeholders were identified using purposive sampling, through existing links with local NHS staff. Participants were also asked to identify other colleagues involved in the NEWS intervention in this area (snowball sampling). We sought to include staff from a range of residential and nursing care homes, particularly with varied experience using NEWS, including long-standing users and users who received the intervention as part of the accelerated roll-out in response to COVID-19. A key NHS employee, known to potential participants, acted as a gatekeeper, sending introductory emails with a participant information sheet. Once consent to be contacted by the research team was secured, a researcher RS, made direct contact by email to invite participation. All individuals approached agreed to take part. Informed consent was secured either electronically or verbally in line with Health Research Authority principles.

All interviews were carried out remotely by telephone or videoconferencing, during May 2020. Development of an interview topic guide (Box 2) was informed by previous research conducted by the team, published literature evaluating the use of NEWS in care homes, and relevant literature and direct communications to the research team describing how care home staff were experiencing the pandemic in the UK. Field notes were taken after interviews.

Box 2. Interview topics

For peer review only - http://bmjopen.bmj.com/site/about/guidelines.xhtml

- Perceived validity of NEWS in care homes
- Experiences of using NEWS before, and during the pandemic
- NEWS training for care homes
- Perceptions on the usefulness of NEWS during the pandemic, including identification of potential cases
- How NEWS influenced decision making and triage, within and outwith care homes
- Variations in use of NEWS during the pandemic

Interviews were carried out by RS or SR (both female Research Associates, doctorates in health services research and medical sociology, and experience of qualitative studies in care homes), audio recorded and transcribed verbatim. All transcripts were anonymised. Interviews lasted 30-60 minutes. Approval was granted by Newcastle University Faculty of Medical Sciences Research Ethics Committee (ref: 2569/2020) and NHS North of England Commissioning Support; HRA approvals were not needed as the work was deemed to be a service evaluation.

Patient and public involvement

Patients and the public were first involved in this study at the idea generation stage, via the Newcastle University Care Home Interest PPI Group (CHIG). The research questions, topic guide, and study design were discussed via email with group members before the study commenced. CHIG members stressed the importance of carrying out interviews remotely and flexibly to time constraints, given the burden of the pandemic on the health and social care sector. Our discussions with this PPI group confirmed that COVID-19 related research in care homes is viewed as a pressing research priority. PPI partners will be consulted on appropriate dissemination strategies.

Data analysis

RS and SR conducted a thematic analysis of the dataset, following the principles of Braun and Clark's six-phase framework[18]. An inductive, data-driven approach was taken to analysis, meaning that the themes generated through our analysis were identified from the data itself, rather than via the use of a pre-existing coding frame.[19] We chose this approach to collect rich data on our topic, using a contextualist approach which "acknowledges the ways individuals make meaning of their experience, and, in turn, the ways the broader social context impinges on those meanings, while retaining focus on the material and other limits of 'reality'." [18] (p. 9) NVivo 11 software was used to aid in data

For peer review only - http://bmjopen.bmj.com/site/about/guidelines.xhtml

management. Data analysis started once the first interview was carried out, and was carried out iteratively, throughout the interviewing period and beyond.

Transcripts were read in full (phase 1 – familiarisation with data), then coded line by line, separately, by RS and SR (phase 2 – generating initial codes). Codes were compared within and between transcripts, using the constant comparative technique[20], to streamline the coding framework and identify themes (phase 3 - search for themes). Emergent themes were discussed within the study team (phase 4 – reviewing themes), and linked together to form a final set of themes and sub-themes (phase 5 – defining themes) which were then situated within each of the three key research questions for the purposes of reporting (phase 6 – writing up analysis). Interviews ceased when no new semantic codes, describing the experiences of staff, were identified from the data (code saturation[19]) as agreed by RS and SR.

Results

We interviewed 17 stakeholders, comprising 10 from the care home sector across 7 care homes, and 7 from the NHS. See Table 1. Of the 7 care homes, 5 provided both residential and nursing care, and 2 provided solely residential care. See Table 2 for care home characteristics. There are no care homes in this area which provide nursing care only. One care home received the accelerated form of the NEWS training and implementation during the COVID-19 pandemic in the UK, the remainder had had NEWS implemented pre-pandemic.

Table 1. Interviewees and their working role

Working role	Sector	n	Description
**Care home manager**<br>***Nursing background***<br>***Non-nursing background***	Care home	7<br>*(3)*<br>*(4)*	Managers oversee the running of the home ensuring that guidelines and regulations are adhered to.
**Care home senior carer/nurse**	Care home	3	Senior carers provide care and limited healthcare to residents and oversee the care provided by junior carers.<br>Care home nurses provide healthcare to residents.
**Director**	NHS	1	Director within a regional NHS Foundation Trust. Provided advice to the implementation team on the use of NEWS

For peer review only - http://bmjopen.bmj.com/site/about/guidelines.xhtml

			within the context of patient safety and reducing avoidable hospital admissions.
**Director**	NHS-related body	1	Supported the implementation team and aided networking and learning between various CCGs and academic institutions.
**NHS nurses**<br>***Specialist Older Persons' Nurse***<br>***Clinical Educator***	NHS	4<br>*(3)*<br>*(1)*	Specialist Nurses visit care homes to provide healthcare to care home residents. They provide a link between the care homes and external services, aiming to prevent avoidable hospital admissions. The Clinical Educator provided training to the care homes as well as ongoing support and monitoring.
**Commissioning of NHS services for older people**	NHS	1	Programme manager with a focus on technological innovation in care settings leading the implementation of NEWS in care homes.
	**Total:**	**17**	

Table 2. Care home characteristics.

Care home	Care provided	Number of beds
**Care home 1**	General care and specialist dementia	~50
**Care home 2**	General care and specialist dementia	~50
**Care home 3**	General care and specialist dementia	~30
**Care home 4**	Predominately specialist dementia	~40
**Care home 5**	Specialist dementia and end of life care	~40
**Care home 6**	Specialist dementia	~40
**Care home 7**	General care and nursing	~30

Higher level themes are organised according to how they address each of the key research questions, which are presented in turn.

For peer review only - http://bmjopen.bmj.com/site/about/guidelines.xhtml

1. **How have care home staff used the NEWS intervention to assess resident illness during the COVID-19 pandemic?**

The NEWS intervention during the pandemic: an adjunct to COVID-19 identification

Many stakeholders described how, prior to the COVID-19 outbreak, the use of the NEWS package had become embedded and habitualised into the everyday routines of care homes, and staff interactions with external healthcare services. Care homes recorded NEWS baseline scores regularly (for routine monitoring and comparison when residents became acutely unwell), except for those near the end of life. A NEWS was frequently requested by community healthcare professionals responding to concerns from care homes, and staff often calculated a NEWS before contacting healthcare professionals.

At the outbreak of the COVID-19 pandemic in the UK and the start of lockdown measures, use of the NEWS by care homes in this area increased substantially. Using NEWS became increasingly embedded into daily care home and NHS practices, and was viewed as a key aspect of the pandemic response in this setting. Care home staff, NHS healthcare professionals and service commissioners shared a belief that measuring NEWS, its component physiological observations, alongside the associated education and support package, have enhanced the response to deteriorating resident health during the pandemic.

Taking vital signs observations and using NEWS with the specific intention of identifying possible COVID-19 infection varied across the care homes in our study. Stakeholders highlighted the power of the NEWS in identifying early illness due to COVID-19 as well as other causes, and the ability of the NEWS intervention to facilitate remote monitoring, thereby decreasing the need for healthcare professionals to visit care homes.

The use of individual physiological observations to identify COVID-19

If COVID-19 was suspected, care home staff frequently measured temperature and oxygen saturations to use as individual physiological measures, as opposed to the NEWS. The use of these physiological observations for identifying possible COVID-19 disease was advocated by a local NHS network. Care home staff appreciated the importance of responding to isolated abnormalities in temperature or oxygen saturations to help identify possible COVID-19, even if the NEWS was not triggering a high level of concern. Stakeholders remained broadly sceptical about the sensitivity and specificity of these clinical observations to accurately help the identification of COVID-19 in this population – feeling that

For peer review only - http://bmjopen.bmj.com/site/about/guidelines.xhtml

they went some way to help, and could prompt further investigation of potential COVID-19, but could not be conclusive.

> *[There are] two main symptoms with our experience, high temperature and low oxygen saturations. We've found in a couple of our residents, […] they had no temperature, and no breathing difficulty, but I couldn't understand - and when we checked their NEWS score we found oxygen saturation was low. So it helps us in the early diagnosis and to do appropriate support for the residents.* Care home manager 1

> *The response to the infection is hugely physiological. So NEWS is a way of prompting you to do physiology. […] I think if you'd asked me six months ago I'd have said 'Actually the NEWS score is more useful than the underlying physiology.' Actually, I'd be tempted to go the other way around now and say 'You do a NEWS score because it makes you look at the physiology and you're going to go, oh my god, look at that oxygen saturation.'* NHS director

Frequency of NEWS recordings and individual observations significantly increased month-on-month from March 2020 onwards, with some care homes taking baseline NEWSs several times a week. Other care homes recorded the NEWS sporadically, suggesting wide variation in the use of NEWS in this area during the UK pandemic. Figure 1 illustrates the increase in NEWS and individual observations recording before and during the pandemic.

FIGURE 1 HERE

Using the NEWS intervention to remotely monitor health

It was felt among care home and NHS healthcare professionals that, prior to the pandemic, NEWS could speed up initial triage and facilitate remote treatment decisions, expediting the process for residents to receive the care they required. Referrals accompanied by a NEWS score reduced the need for clinicians to visit care homes to take physical observations. Identification of early illness was considered to be more efficient with the use of NEWS. Set within this context, it is perhaps not surprising that another positive aspect of the NEWS intervention during the pandemic was to facilitate remote monitoring of residents' health by healthcare professionals. This was believed to have reduced

For peer review only - http://bmjopen.bmj.com/site/about/guidelines.xhtml

unnecessary footfall and thus infection risk, which was a major driver to the accelerated roll out of the NEWS intervention during the pandemic.

> *We had to just take a stand and reflect and say, 'What do we need to do? We know that the predictions [are that COVID-19] is going to come. We're likely to have to go into lockdown. Nobody is going to be able to go into care homes.' So we thought, 'actually NEWS is an important tool in that. […] We just need to respond'* NHS commissioner

> *CCGs have been formally told to set up remote MDT care home rounds, so they are having to put the IT in place. […] You need a digital way of recording [resident] observations, and then you can put in a safety netting process that will allow the care home to get back in touch. Because there's going to be loads of [residents] where you're going to go, 'they're not quite right but I definitely don't want to send them to hospital.'* NHS director

The ability for NHS staff, such as community nurses, to remotely access physiological observations and compare with baselines, was of particular value. The camera facility of the NEWS tablet was also unlocked in response to the pandemic, giving external healthcare professionals the ability to assess and treat wounds remotely.

> *[int: Are there any doctors or GPs who are taking it upon themselves to log on to the [cloud system] and look at the NEWS scores?] Yes, absolutely. [Pre-pandemic] I don't think it was utilised [by them], especially not as much as it could have been, but due to the COVID crisis, everyone across the city now want access to it and it's obviously taken a pandemic to realise how valuable the tool is.* NHS specialist nurse 1

> *I think the biggest change for the [use of the NEWS intervention] at the moment is the pictures. […] For example, if there was a patient who had a skin flap […] We'll say, 'Will you take a photo? Will you upload it so we can see it?' We can physically see that photo and then we can assess the severity, we can assess when the visit should next take place […] it's brilliant for that aspect.* NHS specialist nurse 2

2. What is the experience of care home staff of using the NEWS intervention during the COVID-19 pandemic?

For peer review only - http://bmjopen.bmj.com/site/about/guidelines.xhtml

Empowerment of care home staff with the NHS agenda and language

Using the NEWS intervention to help identify and triage responses to deteriorating resident health was described by stakeholders as empowering. For care home staff, the NEWS intervention represented a helpful link to the NHS. The NEWS intervention represented objective clinical information which care home staff could generally rely upon to back up their intuition that the resident was unwell (termed 'soft signs' e.g. decreased appetite). Having a common clinical language which care home staff could use – or leverage for action, where necessary – was useful to promote integrated working between care homes and the NHS. It also facilitated triage to appropriate services, often negating the need for a primary care healthcare professional to attend in person, which is an important consideration during the pandemic.

Care home staff felt confident that with a NEWS reading, their concerns about deteriorating residents would be taken seriously by external healthcare professionals, as it evidenced their concerns. This was compared positively to care home staff's previous experiences seeking help from primary care, where staff often felt that concerns were not listened to. This feeling of empowerment was capitalised upon by both care homes and local NHS services during the COVID-19 pandemic.

> *When we've rang the likes of home recovery or 111 and we're challenged as to why we're ringing, when we can't really put a finger on it that somebody's not well. Now, this [NEWS] has really aided us to be able to look for those signs, look for those symptoms, using the NEWS score and being able to give better communication across the telephone as to why and what has happened with that particular resident. I know there's been times where we've kind of been brushed off by services. And I'm a big believer in our care staff, they may not be qualified nurses but they know their residents sometimes better than we do. […] Now I feel like we've got this tool now to be able to kind of prove that somewhat as well, and it does help back up what we're saying when it comes to our residents. So it's been great.* Care home manager 2

During the COVID-19 pandemic, care home staff were relied upon to take on clinical procedures and observations usually carried out by visiting NHS professionals. This challenge to traditional role boundaries was welcomed and encouraged by NHS stakeholders, as it met the needs of the NHS to reduce footfall into care homes and limit the number of hospital admissions. In the early days of the pandemic, and without straightforward guidance for care homes, care home staff also welcomed this extension to their roles. They were able to measure and focus on clinical observations – in effect, the

For peer review only - http://bmjopen.bmj.com/site/about/guidelines.xhtml

NEWS intervention facilitated a more objective and decisive response within care homes to tackling a then-new virus.

> *[int: What do you think about the use of NEWS during this pandemic in care homes?] Well I've always loved it. I really love the NEWS tablet and for why, because I think it totally empowers the care staff. […] Care staff, when they say, 'They're just not right. I don't know what's wrong with them, but the person's not right.' Now they've got a little more ammunition to say, 'well actually, such-and-such isn't very well. These are the observations…' and it makes them have more of a voice. That's what I love about it so when they ring the GP and they say, 'Well actually the observations are blah, blah, blah,' and they're almost listened to a little bit more because they've got some sort of clinical information rather than just saying, 'Oh I don't know what's wrong with them.' I think that applies to here and now as well with the pandemic.* NHS specialist nurse 2

3. How has the COVID-19 pandemic influenced the roll out of the NEWS intervention?

Centrality of training relationships and clinical support for accelerated implementation of NEWS during a pandemic

When the pandemic broke out in the UK, the CCG commenced a rapid roll out of the NEWS intervention to the minority of care homes in the area that were waiting for implementation. Care homes already using the NEWS intervention before the pandemic were contacted by the clinical educator to reinforce that support was available. The pandemic highlighted the importance of care homes, particularly those without registered nursing staff, having an engaged and supportive nominated NHS clinical educator.

Training and implementing the NEWS intervention in care homes during the pandemic

Training to support rapid implementation during the pandemic consisted of a short, socially distanced visit from the clinical educator to the care home, training one or two key members of staff in the home rather than a larger body of staff, and without a practical component. This rapid roll out model demonstrated that relationships built during training were integral to ongoing NEWS use and

For peer review only - http://bmjopen.bmj.com/site/about/guidelines.xhtml

understanding. The training constraints related to the pandemic were perceived as having had a major impact on the quality of the rapid implementation and confidence of staff.

> *[During the rapid training], I don't feel like I built great rapport. I don't feel like it instilled that much confidence in them and I haven't really received many phone calls from those homes. Yes they're doing NEWS, but usually the homes that I've really been visibly in, really supported them and built those rapports and stuff up with, I feel like I get more phone calls which is great because they want support which then that in turn means they've picked up something, they're looking for answers and help and they get it. Where those other homes, I would worry that maybe they don't feel as confident to pick up the phone and speak with me because I've raced in, did what I had to do and then left – and it's very difficult to build up rapport over time, and trust.* NHS specialist nurse 1

Ongoing clinical support for care homes during the pandemic

Emphasis was placed on the importance of ongoing clinical support for care homes. In this area, care home staff had access to the clinical educator and a specialist nurse with a specific care home remit. Having these key contacts, who were knowledgeable and approachable, was valued by care home staff and capitalised upon during the COVID-19 pandemic. They were able to build upon long-term relationships with each other, cultivating understanding of each sector and respect for each other's role and remit.

> *This is not just about the technical or the medical aspect, it's often about the relationships that [clinical educator] is then building up. […] I think that methodology has worked because you're consolidating it with a really good grip and really getting [care homes] to be the expert.* NHS commissioner

> *[Clinical educator] is a very approachable person. You can email him, you phone him and in all fairness, we would probably see [clinical educator] at least probably once a week. He'll pop in just to make sure everything is alright and we're well, you know, and if we're having any problems. So that support is continually there.* Care home manager 4

This cross-sector team working helped to legitimise use of NEWS in a non-NHS setting, and smoothed many processes of integrated care over traditional NHS and social care boundaries.

For peer review only - http://bmjopen.bmj.com/site/about/guidelines.xhtml

Discussion

Summary of findings

The roll out of the NEWS intervention in the study area was accelerated by COVID-19. Care home staff and NHS commissioners viewed it as an important part of a combined care home-NHS response to care delivery during the pandemic. The NEWS intervention was used as an adjunct to carer identification of deteriorating resident health, whether due to COVID-19 or other acute illnesses. It was perceived to have facilitated remote decision-making by healthcare and care home staff and minimised footfall in care homes during the pandemic. In relation to possible COVID-19 infection, care home staff reported increased reliance on specific physiological measures such as oxygen saturations, as opposed to the overall NEWS. Care home staff felt empowered by their extended role in the measurement of physiological observations; better able to respond to resident deterioration and communicate their concerns to healthcare professionals. The intervention encompassed training and support from a clinical educator and sharing of clinical measurements and NEWS with health services. Dis-entangling the relative contributions of education, technology and clinical assessment to resident care is challenging.

Comparison with other work

Previous evaluations of the use of NEWS in a non-hospital setting reflect many of our findings, including the benefits of using a common clinical language, and facilitating communication between sectors.[21] The importance of using NEWS as a part of a wider assessment of health, rather than a ritualistic, task-oriented procedure[22], has been stressed by both the UK Royal College of Physicians[9] and those evaluating its use in other settings including primary[13] and community care.[21] The clinician-led training and ongoing support model for the NEWS intervention in place in this area addressed these concerns.

There is some evidence to support the use of NEWS in the identification of and triage of hospital patients with suspected COVID-19.[23 24] However, there have been calls for NEWS to be modified with a more sensitive score for oxygen demand to better account for the development of hypoxic respiratory failure in COVID-19 patients.[25] Evidence for the use of NEWS in community settings during

For peer review only - http://bmjopen.bmj.com/site/about/guidelines.xhtml

the pandemic remains sparse. There is little comparable evidence available to evaluate the utility of NEWS in care homes specifically during the COVID-19 pandemic.

This intervention led to an increase in assessment and monitoring of a vulnerable population of care home residents during a global pandemic. Greater vigilance and attention to physiological measures within care homes may be the critical component, irrespective of the detail of NEWS and adherence to an escalation plan. However, this intervention has a number of features that minimised face to face contacts with residents, which should reduce infection risk for residents and staff. This is important, as a high proportion of care home residents are asymptomatic when they test positive for COVID-19[26], a finding reflected in the experiences of our interviewees. Bluetooth transfer of data from measuring equipment to digital tablet, cloud data storage and the possibility of rapid information transfer to outside agencies, were all of potential benefit during COVID-19. The lack of any requirement to manually input recordings from thermometers and blood pressure monitors was a time saving feature, that promoted accuracy and uptake[27], and in the pandemic, minimised time in close contact with the resident. It is also noteworthy that during COVID-19, staff often completed a subset of measurements, rather than a complete assessment. Selection of what were perceived to be the most important measures (oxygen saturation and temperature) was a modification of NEWS that further reduced staff resident contact. It is also supported by recent evidence[28] that highlights the potential utility of individual oxygen saturation, respiratory rate, and temperature measurements. However, reducing the range of measurements does mean that a NEWS cannot be calculated. Whether the absence of a NEWS score per se over and above the individual measurements has any impact on resident care, is an important question for the future.

From an implementation perspective, the training and support for the NEWS intervention was delivered by an experienced NHS nurse who understood the needs of the sector. In terms of work needed to implement change[27], the organisational burden of planning and implementation for the CCG and NHS clinical educator was minimised as they had already been actively engaged in a revised implementation prior to the pandemic and thus there was a pre-existing awareness and embeddedness of NEWS across the care home sector in the region. Most care home staff in our sample were already familiar with NEWS prior to the pandemic, hence, a shift in roles and identities to take on NEWS related work had already taken place, and only minimal work was needed to engage care home staff in implementation[27] or to change working practices, after the outbreak. This pre-existing familiarity, coupled with the reported ease of use of the NEWS equipment and the support from the wider healthcare system, enhanced the adoption, spread, and scale-up[27] of the NEWS intervention in these care homes during the pandemic.

For peer review only - http://bmjopen.bmj.com/site/about/guidelines.xhtml

Regular, informal contact from a trusted NHS professional to care homes may have been particularly important early in the pandemic, when information was sparse and there were many uncertainties about how best to provide care.[29] The focus in training on identifying 'soft signs' of deterioration was also significant. So called 'soft signs' – a resident having a reduced appetite for a favourite food, for example - are not perceived as measurable or objective. However, they are observed by care home staff, and have their own unique importance.[30] Inclusion within the training could be seen as a validation of care home staff contribution to resident care.

Implementation of change in the care home sector is challenging. Resources are limited and pressures on staff in particular, may limit the potential to engage in new initiatives.[31]Engaging and supporting care homes, with a good understanding of their needs, is critical when implementing change.[16 32] In this case, the intervention was introduced from outside the sector, raising the possibility that it would be perceived as irrelevant or inappropriate for use in care homes. Pre-pandemic and ongoing NHS support for the NEWS intervention legitimised and enhanced its use, reflecting other studies in the care home sector.[33] Widespread changes to working practices during COVID-19 are also likely to have had a major influence on staff readiness to embrace change.

Strengths and limitations

We were able to interview ten care home staff from seven care homes in the locality, and seven NHS staff, despite the pandemic related pressure on the NHS and social care sectors during the interviewing period (May 2020). This represents a strength of our study and highlights the importance that interviewees placed on the topic and expressing their views. However, the views of care home staff working in less senior roles, such as non-senior carers, was less represented due to care home staffing pressures. Our data may not reflect all care home experiences in other areas of the country, particularly those who are not working with NEWS. Similar interventions have been introduced across England, but this model is specific to the study area, which makes comparison with cross-sector interventions in other geographical areas more difficult.

Despite our efforts, we were unable to recruit GPs, which we believe was due to the pressure the pandemic had placed on primary care. The pandemic visiting restrictions in care homes also meant that we were unable to interview any care home residents themselves to explore their experiences during the pandemic and their understanding of care had been influenced by the introduction of the NEWS intervention. Future work should aim to include these groups, so that their views are directly included rather than inferred.

For peer review only - http://bmjopen.bmj.com/site/about/guidelines.xhtml

Conclusion

The NEWS intervention may have a useful role in care homes during the COVID-19 pandemic, enhancing remote working and offering staff some clinical reassurance and structure to their role. However, it is important to acknowledge the paucity of data on NEWS in care homes, despite increasing uptake. Positive staff perceptions from this study now need to be supplemented with data on the impact on resident health and wellbeing, workload, and service utilisation, during the COVID-19 pandemic and beyond. A multidisciplinary consensus on best practice for NEWS use in this sector is required.

For peer review only

Contributors

RS, SR and JA designed the study with JL and BH. RS and SR conducted interviews. RS, SR, and JA analysed data. RS, SR, JA and BH drafted the article, and AR, JL, JG, and RB performed critical revision of the article for important intellectual content. RS is guarantor of the paper. All authors approved the final version to be published.

Data sharing statement

Data are not publicly available.

Word count

5412 words.

Declaration of Conflicts of Interest

The authors declare no conflicts of interest.

Declaration of Funding

This study is funded by the National Institute for Health Research (NIHR), Research for Patient Benefit [award number PB-PG-0418-20034]. JL is funded by the NIHR Applied Research Collaboration North East and North Cumbria (ARC NENC). The views expressed are those of the author(s) and not necessarily those of the NIHR or the Department of Health and Social Care.

References

1. Competition and Markets Authority. Care home market study summary of final report. London, 2017.

For peer review only - http://bmjopen.bmj.com/site/about/guidelines.xhtml

2. Jarrett T. Social care: care home market – structure, issues, and cross-subsidisation. London: House of Commons Library, 2018.
3. Gordon AL, Franklin M, Bradshaw L, et al. Health status of UK care home residents: a cohort study. *Age and Ageing* 2014;43(1):97-103. doi: 10.1093/ageing/aft077
4. Office for National Statistics. Deaths registered weekly in England and Wales, provisional 2020 [Available from: https://www.ons.gov.uk/peoplepopulationandcommunity/birthsdeathsandmarriages/deaths/datasets/weeklyprovisionalfiguresondeathsregisteredinenglandandwales accessed 12th May 2020.
5. Office for National Statistics. Coronavirus (COVID-19) related deaths by occupation, England and Wales: deaths registered up to and including 20 April 2020 2020 [Available from: https://www.ons.gov.uk/releases/covid19relateddeathsbyoccupationenglandandwalesdeathsregistereduptoandincluding20thapril2020 accessed 12th May 2020.
6. World Health Organization. Statement – Invest in the overlooked and unsung: build sustainable people-centred long-term care in the wake of COVID-19 2020 [Available from: http://www.euro.who.int/en/media-centre/sections/statements/2020/statement-invest-in-the-overlooked-and-unsung-build-sustainable-people-centred-long-term-care-in-the-wake-of-covid-19 accessed 12th May 2020.
7. Brainard J, Rushton S, Winters T, et al. Introduction to and spread of COVID-19-like illness in care homes in Norfolk, UK. *Journal of Public Health* 2020 doi: 10.1093/pubmed/fdaa218
8. NHS England. NHS Five Year Forward View. London, 2014.
9. Royal College of Physicians. National Early Warning Score (NEWS) 2: Standardising the assessment of acute-illness severity in the NHS. Updated report of a working party. London: RCP, 2017.
10. West Hampshire Clinical Commissioning Group. RESTORE2: recognise early soft-signs, take observations, respond, escalate 2019 [Available from: https://www.westhampshireccg.nhs.uk/restore2-training-and-resources accessed 08/07/2020.
11. Hodgson P, Cook G, Thompson J, et al. Assessment and clinical decision making of the acutely ill older care home resident: implementation of NEWS in Gateshead Care Homes. Report., 2017.
12. Finnikin S, Hayward G, Wilson F, et al. Are referrals to hospital from out-of-hours primary care associated with National Early Warning Scores? *Emergency Medicine Journal* 2020;37(5):279-85.
13. Greenhalgh T, Treadwell J, Burrow R, et al. Should we use the NEWS (or NEWS2) score when assessing patients with possible COVID-19 in primary care?: Oxford COVID-19 Evidence Service, 2020.
14. Barker RO, Stocker R, Russell S, et al. Distribution of the National Early Warning Score (NEWS) in care home residents. *Age and Ageing* 2019;49(1):141-45. doi: 10.1093/ageing/afz130
15. Hodge S, Thompson C, Gordon AL. National early warning scores in care homes: do policy imperatives reflect a genuine need? *Age and Ageing* 2019;49(1):5-6. doi: 10.1093/ageing/afz149
16. Russell S, Stocker R, Barker RO, et al. Implementation of the National Early Warning Score in UK care homes: a qualitative evaluation. *British Journal of General Practice* 2020;70(700):e793-e800. doi: 10.3399/bjgp20X713069
17. British Geriatrics Society. COVID-19: Managing the COVID-19 pandemic in care homes for older people. Good Practice Guide 2020 [Available from: https://www.bgs.org.uk/resources/covid-19-managing-the-covid-19-pandemic-in-care-homes#anchor-nav-identifying-residents-who-may-have-covid-19-and-how-to-respond accessed 20/04/2020.
18. Braun V, Clarke V. Using thematic analysis in psychology. *Qualitative research in psychology* 2006;3(2):77-101.

For peer review only - http://bmjopen.bmj.com/site/about/guidelines.xhtml

19. Braun V, Clarke V. Reflecting on reflexive thematic analysis. *Qualitative Research in Sport, Exercise and Health* 2019;11(4):589-97.
20. Glaser BG. The constant comparative method of qualitative analysis. *Social problems* 1965;12(4):436-45.
21. Brangan E, Banks J, Brant H, et al. Using the National Early Warning Score (NEWS) outside acute hospital settings: a qualitative study of staff experiences in the West of England. *BMJ Open* 2018;8(10):e022528. doi: 10.1136/bmjopen-2018-022528
22. Massey D, Chaboyer W, Anderson V. What factors influence ward nurses' recognition of and response to patient deterioration? An integrative review of the literature. *Nursing Open* 2017;4(1):6-23. doi: 10.1002/nop2.53
23. Myrstad M, Ihle-Hansen H, Tveita AA, et al. National Early Warning Score 2 (NEWS2) on admission predicts severe disease and in-hospital mortality from Covid-19 – a prospective cohort study. *Scandinavian Journal of Trauma, Resuscitation and Emergency Medicine* 2020;28(1):66. doi: 10.1186/s13049-020-00764-3
24. Gidari A, De Socio GV, Sabbatini S, et al. Predictive value of National Early Warning Score 2 (NEWS2) for intensive care unit admission in patients with SARS-CoV-2 infection. *Infectious Diseases* 2020;52(10):698-704. doi: 10.1080/23744235.2020.1784457
25. Lim NT, Pan D, Barker J. NEWS2 system requires modification to identify deteriorating patients with COVID-19. *Clinical Medicine* 2020;20(4):e133-e34.
26. Graham NSN, Junghans C, Downes R, et al. SARS-CoV-2 infection, clinical features and outcome of COVID-19 in United Kingdom nursing homes. *Journal of Infection* 2020 doi: https://doi.org/10.1016/j.jinf.2020.05.073
27. Greenhalgh T, Wherton J, Papoutsi C, et al. Beyond Adoption: A New Framework for Theorizing and Evaluating Nonadoption, Abandonment, and Challenges to the Scale-Up, Spread, and Sustainability of Health and Care Technologies. *J Med Internet Res* 2017;19(11):e367-e67. doi: 10.2196/jmir.8775
28. Stow D BR, Matthews FE, Hanratty B. . National Early Warning Scores and COVID-19 deaths in care homes: a longitudinal ecological study. *BMJ Open* 2020;In press
29. Spilsbury K, Devi R, Griffiths A, et al. SEeking AnsweRs for Care Homes during the COVID-19 pandemic (COVID SEARCH). *Age and Ageing* 2020 doi: 10.1093/ageing/afaa201
30. Tingström P, Milberg A, Sund-Levander M. Early nonspecific signs and symptoms of infection in institutionalized elderly persons: perceptions of nursing assistants. *Scand J Caring Sci* 2010;24(1):24-31. doi: 10.1111/j.1471-6712.2008.00680.x [published Online First: 2009/12/04]
31. Goodman C, Dening T, Gordon AL, et al. Effective health care for older people living and dying in care homes: a realist review. *BMC Health Services Research* 2016;16:269.
32. Stocker R, Bamford C, Brittain K, et al. Care home services at the vanguard: a qualitative study exploring stakeholder views on the development and evaluation of novel, integrated approaches to enhancing healthcare in care homes. *BMJ Open* 2018;8(3):e017419. doi: 10.1136/bmjopen-2017-017419
33. Gordon AL, Goodman C, Davies SL, et al. Optimal healthcare delivery to care homes in the UK: a realist evaluation of what supports effective working to improve healthcare outcomes. *Age and Ageing* 2018;47(4):595-603. doi: 10.1093/ageing/afx195

For peer review only - http://bmjopen.bmj.com/site/about/guidelines.xhtml

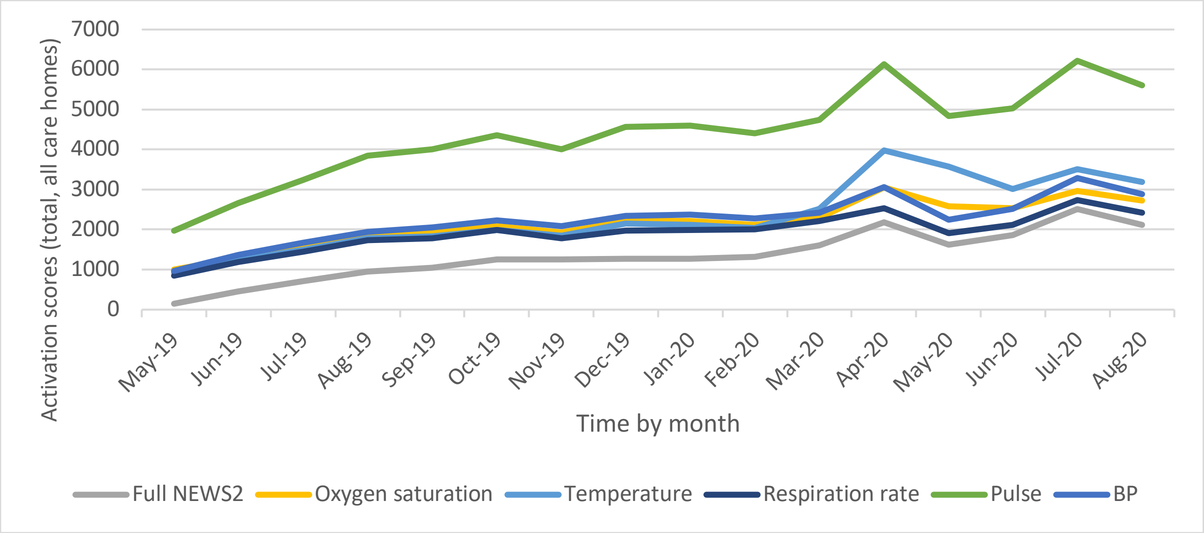

Figure 1 Frequency of NEWS2 and its component measures taken in care homes in the study area

For peer review only

Manuscript: **Remote assessment of care home residents during the COVID-19 pandemic – what is the role of the National Early Warning Score? A qualitative interview study**

Consolidated criteria for reporting qualitative studies (COREQ): 32-item checklist

Developed from:
Tong A, Sainsbury P, Craig J. Consolidated criteria for reporting qualitative research (COREQ): a 32-item checklist for interviews and focus groups. *International Journal for Quality in Health Care*. 2007. Volume 19, Number 6: pp. 349 – 357

No. Item	Guide questions/description	Reported on Page #
**Domain 1: Research team and reflexivity**		
Personal Characteristics		
1. Inter viewer/facilitator	Which author/s conducted the inter view or focus group?	RS and SR, p5
2. Credentials	What were the researcher's credentials? E.g. PhD, MD	PhDs, p5
3. Occupation	What was their occupation at the time of the study?	Research Associates, p5
4. Gender	Was the researcher male or female?	Both female, p5
5. Experience and training	What experience or training did the researcher have?	PhDs and significant experience in care home related qualitative research. P5
Relationship with participants		
6. Relationship established	Was a relationship established prior to study commencement?	No
7. Participant knowledge of the interviewer	What did the participants know about the researcher? e.g. personal goals, reasons for doing the research	Nothing other than the study information
8. Interviewer characteristics	What characteristics were reported about the inter viewer/facilitator? e.g. Bias, assumptions, reasons and interests in the research topic	None relevant

**Domain 2: study design**		
Theoretical framework		
9. Methodological orientation and Theory	What methodological orientation was stated to underpin the study? e.g. grounded theory, discourse analysis, ethnography, phenomenology, content analysis	Phenomenology, p4
Participant selection		
10. Sampling	How were participants selected? e.g. purposive, convenience, consecutive, snowball	Purposive and snowball sampling, p4
11. Method of approach	How were participants approached? e.g. face-to-face, telephone, mail, email	Email, p4
12. Sample size	How many participants were in the study?	17, p5
13. Non-participation	How many people refused to participate or dropped out? Reasons?	No refusals or drop outs, p4
Setting		
14. Setting of data collection	Where was the data collected? e.g. home, clinic, workplace	Home and workplace, p4-5
15. Presence of non-participants	Was anyone else present besides the participants and researchers?	No, p4
16. Description of sample	What are the important characteristics of the sample? e.g. demographic data, date	Information and table on p4-5
Data collection		
17. Interview guide	Were questions, prompts, guides provided by the authors? Was it pilot tested?	Yes, p5
18. Repeat interviews	Were repeat inter views carried out? If yes, how many?	No
19. Audio/visual recording	Did the research use audio or visual recording to collect the data?	Audio recording, p5
20. Field notes	Were field notes made during and/or after the inter view or focus group?	Afterwards, p5
21. Duration	What was the duration of the inter views or focus group?	30-60 minutes, p5
22. Data saturation	Was data saturation discussed?	Yes, p6
23. Transcripts returned	Were transcripts returned to participants for comment and/or correction?	No
**Domain 3: analysis and findings**		
Data analysis		
24. Number of data coders	How many data coders coded the data?	2, p6

For peer review only

25. Description of the coding tree	Did authors provide a description of the coding tree?	No (only themes and sub-themes, p6 onwards)
26. Derivation of themes	Were themes identified in advance or derived from the data?	Derived from the data, p6
27. Software	What software, if applicable, was used to manage the data?	NVivo, p5
28. Participant checking	Did participants provide feedback on the findings?	No
Reporting		
29. Quotations presented	Were participant quotations presented to illustrate the themes/findings? Was each quotation identified? e.g. participant number	Yes, p6 onwards
30. Data and findings consistent	Was there consistency between the data presented and the findings?	Yes
31. Clarity of major themes	Were major themes clearly presented in the findings?	Yes
32. Clarity of minor themes	Is there a description of diverse cases or discussion of minor themes?	Yes

For peer review only
